# Supplementary material for: Phytopathogenic Fungicidal Activity and Mechanism Approach of Three Kinds of Triphenylphosphonium Salts
Source: J Fungi (Basel). 2024 Jun 27;10(7):450. doi: 10.3390/jof10070450 (PMC11278366; doi:10.3390/jof10070450)
Supplement: Supplementary file 1 [file jof-10-00450-s001.zip › jof-3053321-supplementary.pdf]

File S1: ^{1}H NMR, ^{13}C NMR, and HRMS spectrum data of target compounds.

Phytopathogenic Fungicidal Activity and Mechanism Approach of Three Kinds of Triphenylphosphonium Salts

Xuelian Liu [1,2,†], Huihui Liu [1,†], Fahong Yin [1], Yiyi Li [1], Jiazhen Jiang [1], Yumei Xiao [1], Yanhua Wu [1] and Zhaohai Qin [1,*]

[1] College of Science, China Agricultural University, Beijing 100193, China; lxl_nuo@126.com (X.L.); liuhuihui@meibang.cn (H.L.); yinfahong@cau.edu.cn (F.Y.); yiyi.li@cau.edu.cn (Y.L.); jiangjiazhen@cau.edu.cn (J.J.); xiaoyumei@cau.edu.cn (Y.X.); wuyanhua@cau.edu.cn (Y.W.)
[2] School of Pharmaceutical Sciences and Institute of Materia Medica, Xinjiang University, Urumqi 830017, China
* Correspondence: qinzhaohai@263.net; Tel.: +86-10-62732958
† These authors contributed equally to this work.

1. NMR Data for Compounds

Data for **1-1**. Colorless viscous oil, yield 85%. ^{1}H NMR (400 MHz, $CDCl_3$) δ 7.86 – 7.74 (m, 9H), 7.73 – 7.63 (m, 6H), 3.82 – 3.67 (m, 2H), 1.77 – 1.60 (m, 2H), 1.28 – 1.15 (m, 3H). ^{13}C NMR (101 MHz, $CDCl_3$) δ 134.99 (d, *J* = 3.0 Hz), 133.67 (d, *J* = 10.1 Hz), 130.49 (d, *J* = 12.5 Hz), 118.35 (d, *J* = 85.8 Hz), 24.50 (d, *J* = 49.5 Hz), 16.57 (d, *J* = 4.3 Hz), 15.34 (d, *J* = 17.2 Hz). ^{31}P NMR (202 MHz, $CDCl_3$) δ 24.56. HRMS (ESI) *m*/*z* calcd. for $C_{21}H_{22}P$ $[M-Br]^+$ 305.1454, found 305.1451.

Data for **1-2**. Colorless viscous oil, yield 87%. ^{1}H NMR (400 MHz, $CDCl_3$) δ 7.86 – 7.73 (m, 9H), 7.72 – 7.62 (m, 6H), 3.76 – 3.63 (m, 2H), 1.65 – 1.52 (m, 4H), 1.32 – 1.21 (m, 2H), 0.78 (t, *J* = 7.3 Hz, 3H). ^{13}C NMR (101 MHz, $CDCl_3$) δ 135.01 (d, *J* = 3.3 Hz), 133.61 (d, *J* = 10.2 Hz), 130.48 (d, *J* = 12.5 Hz), 118.30 (d, *J* = 85.8 Hz), 32.35 (d, *J* = 15.4 Hz), 22.73 (d, *J* = 49.8 Hz), 22.26 (d, *J* = 4.7 Hz), 22.16, 13.59. ^{31}P NMR (202 MHz, $CDCl_3$) δ 25.11. HRMS (ESI) *m*/*z* calcd. for $C_{23}H_{26}P$ $[M-Br]^+$ 333.1767, found 333.1761.

Data for **1-3**. Colorless viscous oil, yield 81%. ^{1}H NMR (400 MHz, $CDCl_3$) δ 7.88 – 7.75 (m, 9H), 7.74 – 7.66 (m, 6H), 3.85 – 3.70 (m, 2H), 1.68 – 1.55 (m, 4H), 1.27 – 1.16 (m, 4H), 0.87 – 0.75 (m, 3H). ^{13}C NMR (101 MHz, $CDCl_3$) δ 134.99 (d, *J* = 3.0 Hz), 133.74 (d, *J* = 10.2 Hz), 130.50 (d, *J* = 12.5 Hz), 118.49 (d, *J* = 85.7 Hz), 31.37, 30.11 (d, *J* = 15.8 Hz), 22.85 (d, *J* = 49.4 Hz), 22.66 (d, *J* = 4.4 Hz), 22.25, 13.98. ^{31}P NMR (202 MHz, $CDCl_3$) δ 24.19. HRMS (ESI) *m*/*z* calcd. for $C_{24}H_{28}P$ $[M-Br]^+$ 347.1923, found 347.1920.

Data for **1-4**. Colorless viscous oil, yield 75%. ^{1}H NMR (400 MHz, $CDCl_3$) δ 7.86 – 7.75 (m, 9H), 7.73 – 7.65 (m, 6H), 3.80 – 3.68 (m, 2H), 1.65 – 1.53 (m, 4H), 1.28 – 1.12 (m, 6H), 0.80 (t, *J* = 6.9 Hz, 3H). ^{13}C NMR (101 MHz, $CDCl_3$) δ 135.03 (d, *J* = 3.0 Hz), 133.71 (d, *J* = 9.7 Hz), 130.52 (d, *J* = 12.5 Hz), 118.44 (d, *J* = 85.8 Hz), 31.35, 30.39 (d, *J* = 15.5 Hz), 28.87, 22.84 (d, *J* = 49.7 Hz), 22.67 (d, *J* = 4.5 Hz), 22.52, 14.01. ^{31}P NMR (202 MHz, $CDCl_3$) δ 25.16. HRMS (ESI) *m*/*z* calcd. for $C_{25}H_{30}P$ $[M-Br]^+$ 361.2080, found 361.2076.

Data for **1-5**. Colorless viscous oil, yield 65%. ^{1}H NMR (400 MHz, $CDCl_3$) δ 7.87 – 7.75 (m, 9H), 7.73 – 7.66 (m, 6H), 3.81 – 3.67 (m, 2H), 1.66 – 1.55 (m, 4H), 1.28 – 1.13 (m, 8H), 0.81 (t, *J* = 6.8 Hz, 3H). ^{13}C NMR (101 MHz, $CDCl_3$) δ 135.01 (d, *J* = 3.1 Hz), 133.71 (d, *J* = 10.0 Hz), 130.50 (d, *J* = 12.5 Hz), 118.45 (d, *J* = 85.8 Hz), 31.69, 30.42 (d, *J* = 15.5 Hz), 29.17, 28.83, 22.81 (d, *J* = 50.6 Hz), 22.66 (d, *J* = 4.5 Hz), 14.05. ^{31}P NMR (202 MHz, $CDCl_3$) δ 25.06. HRMS (ESI) *m*/*z* calcd. for $C_{26}H_{32}P$ $[M-Br]^+$ 375.2236, found 375.2234.

Data for **1-6**. Colorless viscous oil, yield 72%. ^{1}H NMR (400 MHz, $CDCl_3$) δ 7.85 – 7.72 (m, 9H), 7.72 – 7.62 (m, 6H), 3.74 – 3.52 (m, 2H), 1.67 – 1.46 (m, 4H), 1.29 – 1.07 (m, 10H), 0.86 – 0.74 (m, 3H). ^{13}C NMR (101 MHz, $CDCl_3$) δ 135.04 (d, *J* = 3.0 Hz), 133.57 (d, *J* = 9.6 Hz), 130.49 (d, *J* =

12.5 Hz), 118.23 (d, J = 85.8 Hz), 31.68, 30.37 (d, J = 15.6 Hz), 29.07, 22.73 (d, J = 49.5 Hz), 22.57, 22.52, 14.01. ^{31}P NMR (202 MHz, $CDCl_3$) δ 24.93. HRMS (ESI) m/z calcd. for $C_{27}H_{34}P$ [M-Br]$^+$ 389.2393, found 389.2390.

Data for **1-7**. Colorless viscous oil, yield 67%. ^{1}H NMR (400 MHz, $CDCl_3$) δ 7.88 – 7.76 (m, 9H), 7.74 – 7.66 (m, 6H), 3.90 – 3.65 (m, 2H), 1.66 – 1.57 (m, 4H), 1.28 – 1.15 (m, 12H), 0.84 (t, J = 6.9 Hz, 3H). ^{13}C NMR (101 MHz, $CDCl_3$) δ 134.98 (d, J = 3.0 Hz), 133.73 (d, J = 9.8 Hz), 130.49 (d, J = 12.5 Hz), 118.49 (d, J = 85.8 Hz), 31.83, 30.43 (d, J = 15.4 Hz), 29.48, 29.25, 29.23, 29.19, 22.82 (d, J = 49.4 Hz), 22.70, 22.65, 14.10. ^{31}P NMR (202 MHz, $CDCl_3$) δ 25.08. HRMS (ESI) m/z calcd. for $C_{28}H_{36}P$ [M-Br]$^+$ 403.2549, found 403.2546.

Data for **1-8**. Colorless viscous oil, yield 54%. ^{1}H NMR (400 MHz, $CDCl_3$) δ 7.88 – 7.75 (m, 9H), 7.74 – 7.65 (m, 6H), 3.87 – 3.60 (m, 2H), 1.67 – 1.56 (m, 4H), 1.30 – 1.13 (m, 16H), 0.85 (t, J = 6.8 Hz, 3H). ^{13}C NMR (101 MHz, $CDCl_3$) δ 134.99 (d, J = 3.0 Hz), 133.72 (d, J = 10.0 Hz), 130.49 (d, J = 12.5 Hz), 118.47 (d, J = 85.7 Hz), 31.90, 30.44 (d, J = 15.5 Hz), 29.58, 29.53, 29.32, 29.25, 29.20, 22.83 (d, J = 49.9 Hz), 22.69, 22.68 (d, J = 4.9 Hz), 14.13. ^{31}P NMR (202 MHz, $CDCl_3$) δ 25.19. HRMS (ESI) m/z calcd. for $C_{30}H_{40}P$ [M-Br]$^+$ 431.2862, found 431.2857.

Data for **1-9**. Colorless viscous oil, yield 55%. ^{1}H NMR (400 MHz, $CDCl_3$) δ 7.91 – 7.75 (m, 9H), 7.74 – 7.65 (m, 6H), 3.87 – 3.78 (m, 2H), 1.68 – 1.53 (m, 4H), 1.28 – 1.10 (m, 20H), 0.87 (t, J = 6.8 Hz, 3H). ^{13}C NMR (101 MHz, $CDCl_3$) δ 134.90 (d, J = 3.1 Hz), 133.74 (d, J = 9.9 Hz), 130.43 (d, J = 12.5 Hz), 118.54 (d, J = 85.6 Hz), 31.91, 30.41 (d, J = 15.5 Hz), 29.66, 29.63, 29.62, 29.58, 29.54, 29.34, 29.27, 29.19, 22.82 (d, J = 49.4 Hz), 22.71, 22.68, 14.12. ^{31}P NMR (202 MHz, $CDCl_3$) δ 24.93. HRMS (ESI) m/z calcd. for $C_{32}H_{44}P$ [M-Br]$^+$ 459.3175, found 459.3171.

Data for **1-10**. Colorless viscous oil, yield 57%. ^{1}H NMR (400 MHz, $CDCl_3$) δ 7.89 – 7.75 (m, 9H), 7.73 – 7.66 (m, 6H), 3.85 – 3.74 (m, 2H), 1.66 – 1.56 (m, 4H), 1.27 – 1.14 (m, 24H), 0.86 (t, J = 6.7 Hz, 3H). ^{13}C NMR (101 MHz, $CDCl_3$) δ 134.94 (d, J = 3.1 Hz), 133.71 (d, J = 9.9 Hz), 130.46 (d, J = 12.5 Hz), 118.49 (d, J = 85.7 Hz), 31.91, 30.41 (d, J = 15.5 Hz), 29.68, 29.67, 29.66, 29.65, 29.62, 29.58, 29.53, 29.35, 29.25, 29.19, 22.80 (d, J = 49.5 Hz), 22.68, 22.65, 14.11. ^{31}P NMR (202 MHz, $CDCl_3$) δ 24.87. HRMS (ESI) m/z calcd. for $C_{34}H_{48}P$ [M-Br]$^+$ 487.3488, found 487.3484.

General Synthetic Procedures of Compounds **1A-1C**. A 50 mL solution of acetonitrile, containing 15 mmol of tri-(4-substitutedphenyl)phosphine and 10 mmol of 1-bromoalkane, was refluxed for 24-72 hours. The resulting mixture was then evaporated to dryness. The residue was purified using silica gel column chromatography, with an eluent mixture of petroleum ether, ethyl acetate, and methanol in a ratio of 15:15:1($V/V/V$). This purification method yielded three types of compounds, namely **1A**, **1B**, and **1C**.

Data for 1A-1. Colorless viscous oil, yield 47%. ^{1}H NMR (500 MHz, $CDCl_3$) δ 7.63 (dd, J = 11.9, 8.8 Hz, 6H), 7.15 (dd, J = 8.9, 2.6 Hz, 6H), 3.88 (s, 9H), 3.39 – 3.29 (m, 2H), 1.61 – 1.43 (m, 4H), 1.25 – 1.13 (m, 12H), 0.82 (t, J = 7.0 Hz, 3H). ^{13}C NMR (126 MHz, $CDCl_3$) δ 55.95, 53.44, 31.77, 30.46 (d, J = 15.3 Hz), 29.41, 29.17, 23.77 (d, J = 53.1 Hz), 22.58, 22.53 (d, J = 4.5 Hz), 14.04. ^{31}P NMR (202 MHz, $CDCl_3$) δ 22.80. HRMS (ESI) m/z calcd. for $C_{31}H_{42}O_3P$ [M-Br]$^+$ 493.2866, found 493.2864.

Data for **1A-2**. Colorless viscous oil, yield 45%.^{1}H NMR (500 MHz, $CDCl_3$) δ 7.58 – 7.44 (m, 6H), 7.11 – 6.99 (m, 6H), 3.77 (s, 9H), 3.24 – 3.12 (m, 2H), 1.51 – 1.37 (m, 4H), 1.15 – 1.01 (m, 16H), 0.71 (t, J = 6.9 Hz, 3H). ^{13}C NMR (126 MHz, $CDCl_3$) δ 164.25 (d, J = 4.1 Hz), 135.00 (d, J = 11.8 Hz), 115.90 (d, J = 13.6 Hz), 108.56 (d, J = 93.9 Hz), 55.72, 31.48, 30.15 (d, J = 15.8 Hz), 29.18, 29.12, 28.91, 28.85, 28.79, 23.48 (d, J = 53.3 Hz), 22.27, 22.17 (d, J = 4.2 Hz), 13.74. ^{31}P NMR (202 MHz, $CDCl_3$) δ 22.79. HRMS (ESI) m/z calcd. for $C_{33}H_{46}O_3P$ [M-Br]$^+$ 521.3179, found 521.3179.

Data for **1B-1**. Colorless viscous oil, yield 54%. ^{1}H NMR (500 MHz, $CDCl_3$) δ 7.56 (dd, J = 12.5, 7.9 Hz, 6H), 7.41 (dd, J = 8.2, 3.1 Hz, 6H), 3.46 – 3.32 (m, 2H), 2.40 (s, 9H), 1.58 – 1.46 (m, 4H), 1.21 – 1.09 (m, 12H), 0.77 (t, J = 7.0 Hz, 3H). ^{13}C NMR (126 MHz, $CDCl_3$) δ 145.83 (d, J = 3.1 Hz), 133.00 (d, J = 10.2 Hz), 130.80 (d, J = 13.3 Hz), 114.60 (d, J = 88.4 Hz), 31.36, 30.04 (d, J = 15.1 Hz), 28.99, 28.77, 28.75, 28.70, 22.67 (d, J = 51.2 Hz), 22.18, 22.11 (d, J = 4.4 Hz), 21.44, 13.67. ^{31}P NMR (202 MHz, $CDCl_3$) δ 24.14. HRMS (ESI) m/z calcd. for $C_{31}H_{42}P$ [M-Br]$^+$ 445.3019, found 445.3012.

Data for **1B-2**. Colorless viscous oil, yield 58%. ^{1}H NMR (500 MHz, $CDCl_3$) δ 7.64 – 7.56 (m, 6H), 7.47 – 7.41 (m, 6H), 3.50 – 3.40 (m, 2H), 2.43 (s, 9H), 1.61 – 1.50 (m, 4H), 1.23 – 1.12 (m, 16H),

0.81 (t, J = 6.9 Hz, 3H). ^{13}C NMR (126 MHz, $CDCl_3$) δ 146.23, 133.47 (d, J = 10.2 Hz), 131.19 (d, J = 13.5 Hz), 115.14 (d, J = 88.7 Hz), 31.89, 30.48 (d, J = 15.0 Hz), 29.58, 29.52, 29.32, 29.20, 23.10 (d, J = 51.7 Hz), 22.67, 22.59 (d, J = 4.5 Hz), 21.87, 21.86, 14.12. ^{31}P NMR (202 MHz, $CDCl_3$) δ 24.12. HRMS (ESI) *m/z* calcd. for $C_{33}H_{46}P$ [M-Br]$^+$ 473.3332, found 473.3329.

Data for **1C-1**. Colorless viscous oil, yield 12%. ^{1}H NMR (500 MHz, $CDCl_3$) δ 8.18 (dd, J = 12.6, 8.2 Hz, 6H), 7.90 (dd, J = 8.5, 2.8 Hz, 7H), 4.19 – 4.04 (m, 2H), 1.63 – 1.53 (m, 2H), 1.55 – 1.45 (m, 2H), 1.20 – 1.06 (m, 14H), 0.75 (t, J = 7.0 Hz, 3H). ^{13}C NMR (126 MHz, $CDCl_3$) δ 136.79 (qd, J = 33.8, 3.5 Hz), 134.82 (d, J = 11.3 Hz), 127.65-127.18 (m), 122.66 (q, J = 273.5 Hz), 121.62 (d, J = 84.8 Hz), 31.67, 30.28 (d, J = 16.1 Hz), 29.34, 29.11, 29.06, 22.48, 22.04 (d, J = 46.4 Hz), 13.91. ^{31}P NMR (202 MHz, $CDCl_3$) δ 26.71. HRMS (ESI) *m/z* calcd. for $C_{31}H_{33}F_9P$ [M-Br]$^+$ 607.2171, found 607.2167.

Data for **1C-2**. Colorless viscous oil， yield 8%.^{1}H NMR (500 MHz, $CDCl_3$) δ 8.16 (dd, J = 12.5, 8.2 Hz, 6H), 7.87 (dd, J = 8.5, 2.8 Hz, 6H), 4.17 – 4.06 (m, 2H), 1.59 – 1.42 (m, 4H), 1.17 – 1.04 (m, 16H), 0.73 (t, J = 6.9 Hz, 3H). ^{13}C NMR (126 MHz, $CDCl_3$) δ 136.79 (qd, J = 33.8, 3.5 Hz), 134.82 (d, J = 11.3 Hz), 127.52-127.23 (m), 122.66 (q, J = 273.5 Hz), 121.62 (d, J = 84.8 Hz), 31.67, 30.28 (d, J = 16.1 Hz), 29.34, 29.11, 29.06, 22.48, 22.04 (d, J = 46.4 Hz), 13.91. ^{31}P NMR (202 MHz, $CDCl_3$) δ 25.78. HRMS (ESI) *m/z* calcd. for $C_{33}H_{37}F_9P$ [M-Br]$^+$ 635.2484, found 635.2475.

Data for **2-1**. White powder, mp 235.2 – 236.4 ℃, yield 53%.^{1}H NMR (500 MHz, DMSO-d_6) δ 7.94 – 7.87 (m, 3H), 7.81 – 7.72 (m, 12H), 3.18 (d, J = 14.6 Hz, 2H). ^{13}C NMR (126 MHz, DMSO-d_6) δ 134.82 (d, J = 3.0 Hz), 133.25 (d, J = 10.8 Hz), 130.10 (d, J = 12.7 Hz), 119.92 (d, J = 88.2 Hz), 7.33 (d, J = 55.4 Hz). ^{31}P NMR (202 MHz, DMSO-d_6) δ 22.68. HRMS (ESI) *m/z* calcd. for $C_{20}H_{18}O_2P$ [M-Br]$^+$ 321.1039, found 321.1037.

Data for **2-2**. Colorless viscous oil, yield 46%. ^{1}H NMR (500 MHz, $CDCl_3$) δ 7.85 – 7.64 (m, 15H), 3.79 – 3.69 (m, 2H), 3.08 – 2.97 (m, 2H). ^{13}C NMR (126 MHz, $CDCl_3$) δ 171.47 (d, J = 13.5 Hz), 135.40 (d, J = 2.52 Hz), 133.75 (d, J = 10.08 Hz), 130.80 (d, J = 12.6 Hz), 117.47 (d, J = 86.94 Hz), 27.90, 18.80 (d, J = 55.44 Hz). ^{31}P NMR (202 MHz, $CDCl_3$) δ 25.44 (d, J = 18.7 Hz). HRMS (ESI) *m/z* calcd. for $C_{21}H_{20}O_2P$ [M-Br]$^+$ 335.1195, found 335.1190.

Data for **2-3**. White powder, mp 248.3 – 249.7 ℃, yield 47%. ^{1}H NMR (500 MHz, DMSO-d_6) δ 7.91 – 7.75 (m, 15H), 3.63 (m, 2H), 2.56 – 2.49 (m, 2H), 1.79 – 1.68 (m, 2H). ^{13}C NMR (126 MHz, DMSO-d6) δ 173.29, 134.95 (d, J = 3.3 Hz), 133.58 (d, J = 10.1 Hz), 130.27 (d, J = 12.4 Hz), 118.37 (d, J = 86.2 Hz), 33.64 (d, J = 18.1 Hz), 19.84 (d, J = 51.0 Hz), 17.74 (d, J = 3.6 Hz). Data for **2-4**. Colorless viscous oil, yield 52%. ^{1}H NMR (400 MHz, $CDCl_3$) δ 7.85 - 7.69 (m, 6H), 3.68 – 3.60 (m, 2H), 2.63 (t, J = 6.9 Hz, 2H), 1.94 (p, J = 6.9 Hz, 2H), 1.79 – 1.63 (m, 2H). ^{13}C NMR (101 MHz, $CDCl_3$) δ 175.00, 135.19 (d, J = 3.0 Hz), 133.65 (d, J = 10.1 Hz), 130.62 (d, J = 12.6 Hz), 118.03 (d, J = 85.9 Hz), 33.78, 25.57 (d, J = 17.0 Hz), 22.35 (d, J = 51.2 Hz), 21.56 (d, J = 4.2 Hz). ^{31}P NMR (202 MHz, $CDCl_3$) δ 25.02. HRMS (ESI) *m/z* calcd. for $C_{23}H_{24}O_2P$ [M-Br]$^+$ 363.1508, found 363.1503.

Data for **2-5**. Colorless viscous oil, yield 49%. ^{1}H NMR (500 MHz, DMSO-d_6) δ 7.94 – 7.86 (m, 3H), 7.86 – 7.74 (m, 12H), 3.66 – 3.56 (m, 2H), 2.17 (t, J = 6.8 Hz, 2H), 1.60 – 1.44 (m, 6H). ^{13}C NMR (126 MHz, DMSO-d_6) δ 174.34, 134.90 (d, J = 3.0 Hz), 133.62 (d, J = 10.1 Hz), 130.25 (d, J = 12.4 Hz), 118.56 (d, J = 85.6 Hz), 33.34, 29.34 (d, J = 16.9 Hz), 23.70, 21.59 (d, J = 4.3 Hz), 20.15 (d, J = 50.0 Hz). ^{31}P NMR (202 MHz, $CDCl_3$) δ 25.16. HRMS (ESI) *m/z* calcd. for $C_{24}H_{26}O_2P$ [M-Br]$^+$ 377.1662, found 377.1665.

Data for **2-6**. White powder, mp 189.5 – 190.2 ℃, yield 49%. ^{1}H NMR (500 MHz, DMSO-d_6) δ 7.93 - 7.68 (m, 15H), 3.64 – 3.55 (m, 2H), 2.17 (t, J = 7.3 Hz, 2H), 1.61 – 1.38 (m, 6H), 1.36 – 1.23 (m, 2H). ^{13}C NMR (126 MHz, DMSO-d_6) δ 174.36, 134.85 (d, J = 3.1 Hz), 133.57 (d, J = 10.1 Hz), 130.21 (d, J = 12.3 Hz), 118.56 (d, J = 85.9 Hz), 33.48, 29.48 (d, J = 16.7 Hz), 27.64, 24.10, 21.63 (d, J = 4.5 Hz), 20.17 (d, J = 49.8 Hz). ^{31}P NMR (202 MHz, DMSO-d_6) δ 24.07. HRMS (ESI) *m/z* calcd. for $C_{25}H_{28}O_2P$ [M-Br]$^+$ 391.1921, found 391.1825.

Data for **2-7**. White powder m.p.114.1-115.2 ℃， yield 53%. ^{1}H NMR (500 MHz, $CDCl_3$) δ 7.78 – 7.69 (m, 9H), 7.68 – 7.62 (m, 6H), 3.57 – 3.50 (m, 2H), 2.31 (t, J = 7.3 Hz, 2H), 1.63 – 1.45 (m, 6H), 1.30 – 1.14 (m, 4H). ^{13}C NMR (126 MHz, DMSO-d_6) δ 174.42, 134.85 (d, J = 2.9 Hz), 133.56 (d, J = 10.2 Hz), 130.21 (d, J = 12.4 Hz), 118.57 (d, J = 85.7 Hz), 33.56, 29.62 (d, J = 16.6 Hz), 28.18, 27.76,

24.29, 21.69 (d, J = 4.3 Hz), 20.14 (d, J = 49.7 Hz). ^{31}P NMR (202 MHz, $CDCl_3$) δ 25.02. HRMS (ESI) m/z calcd. for $C_{26}H_{30}O_2P$ [M-Br]$^+$ 405.1978, found 405.1974.

Data for **2-8**. Colorless viscous oil，yield 47%. ^{1}H NMR (500 MHz, $CDCl_3$) δ 7.81 – 7.67 (m, 9H), 7.65-7.61 (m, 6H), 3.52 – 3.41 (m, 2H), 2.20 (t, J = 7.4 Hz, 2H), 1.53-1.49 (m, 4H), 1.46 – 1.39 (m, 2H), 1.19 – 1.08 (m, 6H). ^{13}C NMR (126 MHz, $CDCl_3$) δ 176.94, 135.05 (d, J = 2.6 Hz), 133.45 (d, J = 10.2 Hz), 130.49 (d, J = 12.5 Hz), 118.03 (d, J = 85.8 Hz), 34.34, 30.00 (d, J = 15.6 Hz), 28.44, 28.42, 28.39, 24.49, 22.51 (d, J = 47.5 Hz), 22.29. ^{31}P NMR (202 MHz, $CDCl_3$) δ 25.14 (s). HRMS (ESI) m/z calcd for $C_{27}H_{32}O_2P$ [M-Br]$^+$ 419.2134, found 419.2131.

Data for **2-9**. Colorless viscous oil，yield 39%. ^{1}H NMR (400 MHz, $CDCl_3$) δ 7.83 – 7.47 (m, 15H), 3.68 – 3.15 (m, 2H), 2.16 (t, J = 7.4 Hz, 2H), 1.56 – 1.30 (m, 6H), 1.14 – 0.93 (m, 8H). ^{13}C NMR (126 MHz, $CDCl_3$) δ 177.09, 135.09 (d, J = 3.2 Hz), 133.49 (d, J = 9.8 Hz), 130.52 (d, J = 12.5 Hz), 118.09 (d, J = 85.9 Hz), 34.40, 30.10 (d, J = 15.8 Hz), 28.66, 28.59, 24.59, 22.55 (d, J = 44.1 Hz), 22.33. ^{31}P NMR (202 MHz, $CDCl_3$) δ 24.94. HRMS (ESI) m/z calcd. for $C_{28}H_{34}O_2P$ [M-Br]$^+$ 433.2291, found 433.2289.

Data for **2-10**. Colorless viscous oil，yield 37%. ^{1}H NMR (400 MHz, $CDCl_3$) δ 7.83 - 7.76 (m, 15H), 3.71 – 3.47 (m, 2H), 2.33 (t, J = 7.1 Hz, 2H), 1.73 – 1.44 (m, 6H), 1.25-1.18 (m, 10H). ^{13}C NMR (126 MHz, $CDCl_3$) δ 177.41, 135.45 (d, J = 3.0 Hz), 133.41 (d, J = 9.9 Hz), 130.43 (d, J = 12.3 Hz), 118.01 (d, J = 85.8), 34.39, 30.15 (d, J = 15.7 Hz), 28.87, 28.74, 28.69, 28.64, 24.60, 22.53 (d, J = 42.2 Hz), 22.34, 22.30. ^{31}P NMR (202 MHz, $CDCl_3$) δ 25.19. HRMS (ESI) m/z calcd. for $C_{29}H_{36}O_2P$ [M-Br]$^+$ 447.2447, found 447.2445.

Data for **2-11**. Colorless viscous oil，yield 43%.^{1}H NMR (500 MHz, DMSO-d_6) δ 7.93 – 7.88 (m, 3H), 7.84 – 7.75 (m, 12H), 3.64 – 3.54 (m, 2H), 2.18 (t, J = 7.4 Hz, 2H), 1.57 – 1.42 (m, 6H), 1.30 – 1.14 (m, 12H). ^{13}C NMR (101 MHz, $CDCl_3$) δ 177.45, 135.16 (d, J = 3.0 Hz), 133.81 (d, J = 10.2 Hz), 130.64 (d, J = 12.5 Hz), 118.53 (d, J = 85.8 Hz), 34.35, 30.47, 30.31, 29.02, 28.96, 28.94, 28.84, 28.75, 24.77, 22.84 (d, J = 50.0 Hz), 22.74 (d, J = 4.5 Hz). ^{31}P NMR (202 MHz, $CDCl_3$) δ 25.11. HRMS (ESI) m/z calcd. for $C_{30}H_{38}O_2P$ [M-Br]$^+$ 461.2604, found 461.2601.

Data for **2-12**. White powder, mp 92.6 – 93.6 ℃, yield 49%, yield 62%. ^{1}H NMR (500 MHz, DMSO-d_6) δ 7.93 – 7.74 (m, 15H), 3.63 – 3.54 (m, 2H), 2.18 (t, J = 7.3 Hz, 2H), 1.58 – 1.42 (m, 6H), 1.30 – 1.18 (m, 18H). ^{13}C NMR (126 MHz, DMSO-d_6) δ 174.49, 134.82 (d, J = 2.9 Hz), 133.56 (d, J = 10.2 Hz), 130.19 (d, J = 12.4 Hz), 118.57 (d, J = 85.7 Hz), 33.70, 29.75 (d, J = 16.5 Hz), 28.97, 28.93, 28.87, 28.83, 28.71, 28.66, 28.52, 28.07, 24.48, 21.71 (d, J = 4.4 Hz), 20.17 (d, J = 49.7 Hz). ^{31}P NMR (202 MHz, DMSO-d_6) δ 24.09. HRMS (ESI) m/z calcd. for $C_{33}H_{44}O_2P$ [M-Br]$^+$ 503.3073, found 503.3058.

Data for **2-13**. White powder, mp 109.1 – 111.1℃, yield 57%, ^{1}H NMR (500 MHz, DMSO-d_6) δ 7.90 (td, J = 7.3, 1.8 Hz, 3H), 7.85 – 7.74 (m, 12H), 3.64 – 3.55 (m, 2H), 2.18 (t, J = 7.4 Hz, 2H), 1.58 – 1.40 (m, 6H), 1.31 – 1.14 (m, 21H). ^{13}C NMR (126 MHz, DMSO-d_6) δ 174.50, 134.83 (d, J = 3.1 Hz), 133.56 (d, J = 10.1 Hz), 130.19 (d, J = 12.4 Hz), 118.57 (d, J = 85.5 Hz), 33.70, 29.76 (d, J = 16.4 Hz), 28.98, 28.97, 28.95, 28.93, 28.87, 28.83, 28.71, 28.66, 28.52, 28.06, 24.48, 21.71 (d, J = 4.5 Hz), 20.16 (d, J = 49.9 Hz). ^{31}P NMR (202 MHz, DMSO-d_6) δ 24.08. HRMS (ESI) m/z calcd. for $C_{34}H_{46}OP$ [M-Br]$^+$ 517.3230, found 517.3219.

Data for **2-14**. White powder, mp 112.9 – 113.7 ℃, yield 63%. ^{1}H NMR (500 MHz, DMSO-d_6) δ 7.97 (td, J = 7.1, 1.8 Hz, 3H), 7.92 – 7.80 (m, 12H), 3.69 – 3.60 (m, 2H), 2.24 (t, J = 7.4 Hz, 2H), 1.61 – 1.47 (m, 6H), 1.36 – 1.24 (m, 22H). ^{13}C NMR (126 MHz, DMSO-d_6) δ 174.48, 134.83 (d, J = 2.9 Hz), 133.56 (d, J = 10.1 Hz), 130.19 (d, J = 12.4 Hz), 118.57 (d, J = 85.5 Hz), 33.70, 29.76 (d, J = 16.6 Hz), 28.99, 28.96, 28.93, 28.87, 28.84, 28.71, 28.67, 28.52, 28.07, 24.48, 21.71 (d, J = 4.5 Hz), 20.17 (d, J = 49.7 Hz). ^{31}P NMR (202 MHz, DMSO-d_6) δ 24.09. HRMS (ESI) m/z calcd. for $C_{35}H_{48}O_2P$ [M-Br]$^+$ 531.3386, found 531.3378.

Data for **2-15.** White powder, mp 124.0 – 125.4 ℃, yield 58%. ^{1}H NMR (500 MHz, DMSO-d_6) δ 7.91 (td, J = 7.1, 1.7 Hz, 3H), 7.85 – 7.75 (m, 12H), 3.65 – 3.55 (m, 2H), 2.18 (t, J = 7.3 Hz, 2H), 1.58 – 1.40 (m, 6H), 1.31 – 1.13 (m, 28H). ^{13}C NMR (126 MHz, DMSO-d_6) δ 174.43, 134.83 (d, J = 3.2 Hz), 133.55 (d, J = 10.1 Hz), 130.19 (d, J = 12.4 Hz), 118.57 (d, J = 85.5 Hz), 33.66, 29.77 (d, J = 16.5 Hz), 29.00, 28.96, 28.95, 28.88, 28.85, 28.71, 28.68, 28.52, 28.08, 24.47, 21.72 (d, J = 4.4 Hz), 20.17 (d, J = 49.7 Hz). ^{31}P NMR (202 MHz, DMSO-d_6) δ 24.08. HRMS (ESI) m/z calcd. for $C_{38}H_{54}O_2P$ [M-Br]$^+$ 573.3856, found 573.3859.

Data for **2-12′**. Viscous oil, yield 60%. ^{1}H NMR (500 MHz, $CDCl_3$) δ 7.82 – 7.70 (m, 9H), 7.68 – 7.60 (m, 6H), 3.78 – 3.69 (m, 2H), 3.59 (s, 3H), 2.23 (t, *J* = 7.5 Hz, 2H), 1.59 – 1.49 (m, 6H), 1.26- 1.11 (m, 18H). ^{13}C NMR (126 MHz, $CDCl_3$) δ 174.38, 134.96 (d, *J* = 3.1 Hz), 133.73 (d, *J* = 9.9 Hz), 130.48 (d, *J* = 12.5 Hz), 118.48 (d, *J* = 85.6 Hz), 51.45, 34.13, 30.44 (d, *J* = 15.7 Hz), 29.57, 29.55, 29.53, 29.43, 29.28, 29.25, 29.20, 29.14, 24.96, 22.82 (d, *J* = 49.4 Hz), 22.69 (d, *J* = 4.8 Hz). ^{31}P NMR (202 MHz, $CDCl_3$) δ 24.28. HRMS (ESI) m/z calcd. for $C_{34}H_{46}O_2P$ [M-Br]$^+$ 517.3230, found 517.3224.

Data for **3-1**. Colorless viscous oil，yield 68%. ^{1}H NMR (400 MHz, $CDCl_3$) δ 7.83 – 7.70 (m, 9H), 7.69 – 7.59 (m, 6H), 4.04 (dt, *J* = 18.6, 6.0 Hz, 2H), 3.78 (dt, *J* = 12.1, 6.1 Hz, 2H). ^{13}C NMR (101 MHz, $CDCl_3$) δ 134.88 (d, *J* = 3.2 Hz), 133.80 (d, *J* = 10.3 Hz), 130.27 (d, *J* = 12.9 Hz), 118.59 (d, *J* = 86.5 Hz), 55.38 (d, *J* = 5.2 Hz), 27.08 (d, *J* = 50.4 Hz). ^{31}P NMR (202 MHz, $CDCl_3$) δ 25.70. HRMS (ESI) *m/z* calcd. for $C_{20}H_{20}OP$ [M-Br]$^+$ 307.1246, found 307.1245.

Data for **3-2**. Colorless viscous oil，yield 59%. ^{1}H NMR (400 MHz, $CDCl_3$) δ 7.81 – 7.72 (m, 9H), 7.71 – 7.64 (m, 6H), 3.86 – 3.78 (m, 2H), 3.78 – 3.69 (m, 2H), 1.89 – 1.75 (m, 2H). ^{13}C NMR (101 MHz, $CDCl_3$) δ 135.07 (d, *J* = 3.0 Hz), 133.50 (d, *J* = 9.9 Hz), 130.55 (d, *J* = 12.5 Hz), 118.36 (d, *J* = 86.1 Hz), 60.33 (d, *J* = 16.7 Hz), 25.85 (d, *J* = 4.3 Hz), 20.17 (d, *J* = 52.7 Hz). ^{31}P NMR (202 MHz, $CDCl_3$) δ 25.66. HRMS (ESI) *m/z* calcd. for $C_{21}H_{22}OP$ [M-Br]$^+$ 321.1403, found 321.1400.

Data for **3-3**. White powder, mp 238.9 – 240.2 ℃, yield 43%.^{1}H NMR (500 MHz, DMSO-*d*$_6$) δ 7.95 – 7.88 (m, 3H), 7.85 – 7.75 (m, 12H), 3.86 – 3.73 (m, 2H), 1.87 – 1.73 (m, 6H). ^{13}C NMR (126 MHz, DMSO-d6) δ 134.97 (d, *J* = 3.1 Hz), 133.63 (d, *J* = 10.2 Hz), 130.31 (d, *J* = 12.4 Hz), 118.34 (d, *J* = 85.9 Hz), 22.56 (dd, *J* = 18.5, 3.6 Hz), 22.39, 19.97 (d, *J* = 50.7 Hz). ^{31}P NMR (202 MHz, DMSO-*d*$_6$) δ 23.82. HRMS (ESI) *m/z* calcd. for $C_{22}H_{24}OP$ [M-Br]$^+$ 335.1559, found 335.1556.

Data for **3-4**. White powder, mp 195.1 – 196.2℃, yield 54%. ^{1}H NMR (500 MHz, DMSO-*d*$_6$) δ 7.92– 7.74 (m, 15H), 3.66 – 3.57 (m, 2H), 3.34 (t, *J* = 5.7 Hz, 2H), 1.59 – 1.39 (m, 6H). ^{13}C NMR (126 MHz, DMSO-*d*$_6$) δ 134.82 (d, *J* = 3.0 Hz), 133.57 (d, *J* = 10.2 Hz), 130.19 (d, *J* = 12.4 Hz), 118.57 (d, *J* = 85.7 Hz), 60.19, 31.47, 26.50 (d, *J* = 17.0 Hz), 21.71 (d, *J* = 4.4 Hz), 20.27 (d, *J* = 49.7 Hz). ^{31}P NMR (202 MHz, DMSO-*d*$_6$) δ 24.04. HRMS (ESI) *m/z* calcd. for $C_{23}H_{26}OP$ [M-Br]$^+$ 349.1716, found 349.1712.

Data for **3-5**. White powder, mp 135.7 – 136.2℃, yield 57%. ^{1}H NMR (400 MHz, $CDCl_3$) δ 7.88 – 7.75 (m, 9H), 7.74 – 7.66 (m, 6H), 3.84 – 3.68 (m, 2H), 3.66 – 3.58 (m, 2H), 1.74 – 1.59 (m, 4H), 1.56 – 1.37 (m, 4H). ^{13}C NMR (101 MHz, $CDCl_3$) δ 135.02 (d, *J* = 3.2 Hz), 133.74 (d, *J* = 9.7 Hz), 130.54 (d, *J* = 12.5 Hz), 118.47 (d, *J* = 85.8 Hz), 61.57, 31.91, 29.48 (d, *J* = 16.1 Hz), 24.79, 22.53 (d, *J* = 49.9 Hz), 22.40 (d, *J* = 4.5 Hz). ^{31}P NMR (202 MHz, DMSO-*d*$_6$) δ 24.05. HRMS (ESI) *m/z* calcd. for $C_{24}H_{28}OP$ [M-Br]$^+$ 363.1872, found 363.1870.

Data for **3-6**. Colorless viscous oil，yield 47%. ^{1}H NMR (400 MHz, $CDCl_3$) δ 7.87 – 7.76 (m, 9H), 7.74 – 7.67 (m, 6H), 3.80 – 3.67 (m, 2H), 3.60 (t, *J* = 6.3 Hz, 2H), 1.71 – 1.59 (m, 4H), 1.53 (p, *J* = 6.6 Hz, 2H), 1.39 – 1.28 (m, 4H). ^{13}C NMR (101 MHz, $CDCl_3$) δ 135.02 (d, *J* = 3.0 Hz), 133.73 (d, *J* = 10.2 Hz), 130.53 (d, *J* = 12.5 Hz), 118.46 (d, *J* = 85.8 Hz), 62.14, 32.10, 29.98 (d, *J* = 16.1 Hz), 28.20, 25.08, 22.68 (d, *J* = 49.8 Hz), 22.32 (d, *J* = 4.5 Hz). ^{31}P NMR (202 MHz, $CDCl_3$) δ 25.17. HRMS (ESI) *m/z* calcd. for $C_{25}H_{30}OP$ [M-Br]$^+$ 377.2029, found 377.2027.

Data for **3-7**. White powder, mp 96.5 – 97.5 ℃, yield 65%. ^{1}H NMR (500 MHz, DMSO-*d*$_6$) δ 7.90 (m, 3H), 7.85 – 7.73 (m, 12H), 4.35 (t, *J* = 5.1 Hz, 1H), 3.64 – 3.55 (m, 2H), 3.36 – 3.31 (m, 2H), 1.56 – 1.34 (m, 6H), 1.31 – 1.16 (m, 6H). ^{13}C NMR (126 MHz, DMSO-*d*$_6$) δ 134.83 (d, *J* = 2.9 Hz), 133.56 (d, *J* = 10.1 Hz), 130.20 (d, *J* = 12.4 Hz), 118.57 (d, *J* = 85.8 Hz), 60.62, 32.39, 29.74 (d, *J* = 16.5 Hz), 28.58, 28.12, 25.32, 21.71 (d, *J* = 4.4 Hz), 20.16 (d, *J* = 49.7 Hz). ^{31}P NMR (202 MHz, DMSO-*d*$_6$) δ 24.09. HRMS (ESI) *m/z* calcd. for $C_{26}H_{32}OP$ [M-Br]$^+$ 391.2185, found 391.2176.

Data for **3-8**. Colorless viscous oil, yield 39%. ^{1}H NMR (500 MHz, DMSO-*d*$_6$) δ 7.94 – 7.86 (m, 3H), 7.85 – 7.74 (m, 12H), 4.34 (t, *J* = 5.1 Hz, 1H), 3.64 – 3.54 (m, 2H), 3.38 – 3.34 (m, 2H), 1.57 – 1.49 (m, 2H), 1.48 – 1.41 (m, 2H), 1.41 – 1.33 (m, 2H), 1.30 – 1.17 (m, 8H). ^{13}C NMR (126 MHz, $CDCl_3$) δ 134.91 (d, *J* = 3.1 Hz), 133.29 (d, *J* = 10.1 Hz), 130.34 (d, *J* = 12.5 Hz), 117.88 (d, *J* = 85.9 Hz), 61.94, 32.29, 29.97 (d, *J* = 15.5 Hz), 28.74, 28.64, 28.47, 25.30, 22.40 (d, *J* = 46.4 Hz), 22.18. ^{31}P NMR (202 MHz, $CDCl_3$) δ 25.18. HRMS (ESI) *m/z* calcd. for $C_{27}H_{34}OP$ [M-Br]$^+$ 405.2342, found 405.2340.

Data for **3-9**. White powder, mp 90.1 – 91.2 ℃, yield 60%.^{1}H NMR (500 MHz, DMSO-d_6) δ 7.93 – 7.88 (m, 3H), 7.85 – 7.75 (m, 12H), 4.35 (t, *J* = 5.1 Hz, 1H), 3.63 – 3.54 (m, 2H), 3.38 – 3.34 (m, 2H), 1.56 – 1.35 (m, 6H), 1.30 – 1.18 (m, 10H). ^{13}C NMR (126 MHz, DMSO-d_6) δ 134.84 (d, *J* = 3.0 Hz), 133.56 (d, *J* = 10.1 Hz), 130.20 (d, *J* = 12.1 Hz), 118.57 (d, *J* = 85.5 Hz), 60.65, 32.47, 29.76 (d, *J* = 16.5 Hz), 28.88, 28.84, 28.63, 28.06, 25.46, 21.71 (d, *J* = 4.4 Hz), 20.16 (d, *J* = 49.7 Hz). ^{31}P NMR (202 MHz, DMSO-d_6) δ 24.08. HRMS (ESI) *m/z* calcd. for $C_{28}H_{36}OP$ [M-Br]$^+$ 419.2498, found 419.2496.

Data for **3-10**. Colorless viscous oil, yield 45%. ^{1}H NMR (400 MHz, $CDCl_3$) δ 7.88 – 7.74 (m, 9H), 7.74 – 7.65 (m, 6H), 3.82 – 3.67 (m, 2H), 3.61 (t, *J* = 6.6 Hz, 2H), 1.67 – 1.56 (m, 4H), 1.56 – 1.48 (m, 2H), 1.33 – 1.16 (m, 12H). ^{13}C NMR (101 MHz, $CDCl_3$) δ 135.02 (d, *J* = 3.0 Hz), 133.71 (d, *J* = 10.2 Hz), 130.51 (d, *J* = 12.5 Hz), 118.44 (d, *J* = 85.8 Hz), 62.87, 32.64, 30.36 (d, *J* = 15.4 Hz), 29.30, 29.20, 29.08, 29.03, 25.63, 22.80 (d, *J* = 49.7 Hz), 22.69, 22.64 . ^{31}P NMR (202 MHz, $CDCl_3$) δ 25.17. HRMS (ESI) *m/z* calcd. for $C_{29}H_{38}OP$ [M-Br]$^+$ 433.2655, found 433.2653.

Data for **3-11**. White powder, mp 92.6 – 93.8℃, yield 61%. ^{1}H NMR (400 MHz, Chloroform-*d*) δ 7.82 – 7.71 (m, 9H), 7.71 – 7.62 (m, 6H), 3.70 – 3.57 (m, 2H), 3.55 (t, *J* = 6.7 Hz, 2H), 1.64 – 1.51 (m, 4H), 1.47 (q, *J* = 7.0 Hz, 2H), 1.27 – 1.11 (m, 14H). ^{13}C NMR (126 MHz, DMSO-d_6) δ 134.83 (d, *J* = 3.0 Hz), 133.56 (d, *J* = 9.8 Hz), 130.19 (d, *J* = 12.4 Hz), 118.57 (d, *J* = 85.5 Hz), 60.66, 32.49, 29.75 (d, *J* = 16.6 Hz), 29.03, 28.90, 28.83, 28.66, 28.07, 25.47, 21.71 (d, *J* = 4.4 Hz), 20.17 (d, *J* = 49.7 Hz). ^{31}P NMR (202 MHz, DMSO-d_6) δ 24.08. HRMS (ESI) *m/z* calcd. for $C_{30}H_{40}OP$ [M-Br]$^+$ 447.2811, found 447.2810.

Data for **3-12**. White powder, mp 109.2 – 110.8 ℃, yield 58%.^{1}H NMR (500 MHz, DMSO-d_6) δ 7.93 – 7.88 (m, 3H), 7.84 – 7.75 (m, 12H), 4.34 (t, *J* = 5.1 Hz, 1H), 3.63 – 3.53 (m, 2H), 3.39 – 3.36 (m, 2H), 1.55 – 1.36 (m, 6H), 1.30 – 1.18 (m, 18H). ^{13}C NMR (126 MHz, DMSO-d_6) δ 134.82 (d, *J* = 2.9 Hz), 133.55 (d, *J* = 10.1 Hz), 130.18 (d, *J* = 12.4 Hz), 118.56 (d, *J* = 85.7 Hz), 60.66, 32.50, 29.75 (d, *J* = 16.6 Hz), 29.05, 28.99, 28.96, 28.93, 28.92, 28.83, 28.66, 28.07, 25.47, 21.71 (d, *J* = 4.3 Hz), 20.17 (d, *J* = 49.7 Hz). ^{31}P NMR (202 MHz, DMSO-d_6) δ 24.09. HRMS (ESI) *m/z* calcd. for $C_{32}H_{44}OP$ [M-Br]$^+$ 475.3124, found 475.3131.

Data for **3-13**. White powder, mp 107.8 – 108.7 ℃, yield 47%.^{1}H NMR (500 MHz, DMSO-d_6) δ 7.92 – 7.74 (m, 15H), 4.38 (t, *J* = 5.1 Hz, 1H), 3.61 – 3.54 (m, 2H), 3.39 – 3.35 (m, 2H), 1.56 – 1.37 (m, 6H), 1.30 – 1.17 (m, 22H). ^{13}C NMR (126 MHz, DMSO-d_6) δ 134.83 (d, *J* = 2.9 Hz), 133.55 (d, *J* = 10.1 Hz), 130.19 (d, *J* = 12.4 Hz), 118.57 (d, *J* = 85.3 Hz), 60.67, 32.51, 29.76 (d, *J* = 16.6 Hz), 29.06, 28.99, 28.94, 28.92, 28.84, 28.67, 28.08, 25.48, 21.71 (d, *J* = 4.4 Hz), 20.16 (d, *J* = 49.7 Hz). ^{31}P NMR (202 MHz, DMSO-d_6) δ 24.08. HRMS (ESI) *m/z* calcd. for $C_{34}H_{48}OP$ [M-Br]$^+$ 503.3437, found 503.3440

Data for **3-11'**. Colorless oil, yield 64%. ^{1}H NMR (500 MHz, $CDCl_3$) δ 7.87 – 7.80 (m, 9H), 7.77 – 7.71 (m, 6H), 4.04 (t, *J* = 6.8 Hz, 2H), 3.72 – 3.63 (m, 2H), 2.04 (s, 3H), 1.66 – 1.57 (m, 6H), 1.33 – 1.18 (m, 14H). ^{13}C NMR (126 MHz, $CDCl_3$) δ 171.06, 134.93 (d, *J* = 3.1 Hz), 133.41 (d, *J* = 9.8 Hz), 130.37 (d, *J* = 12.5 Hz), 118.02 (d, *J* = 85.9 Hz), 64.41, 30.25 (d, *J* = 15.4 Hz), 29.22, 29.21, 28.97, 28.34, 25.65, 22.61 (d, *J* = 44.0 Hz), 22.39, 20.86. ^{31}P NMR (202 MHz, $CDCl_3$) δ 24.29. HRMS (ESI) *m/z* calcd. for $C_{32}H_{42}O_2P$ [M-Br]$^+$ 489.2917, found 489.2919.

2. NMR SPECTRA

Figure S1. Spectra of compound **1-X**, **2-X** and **3-X**.

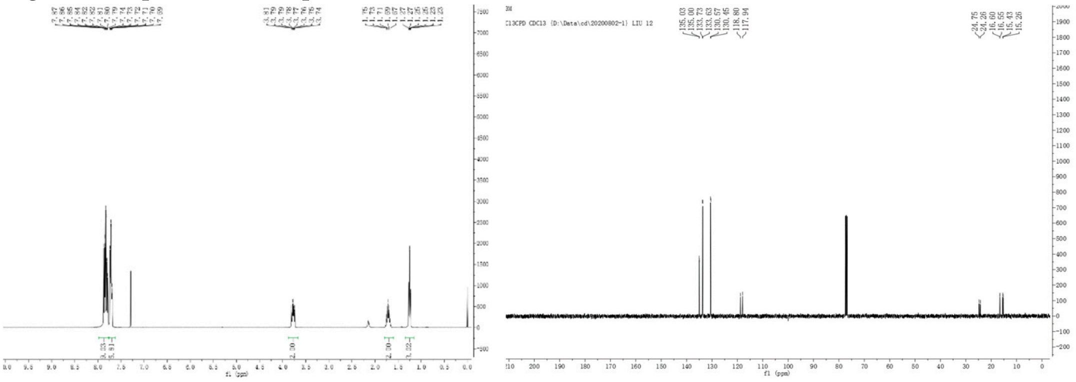

^{1}HNMR of **1-1** (C_2CH_3)　　^{13}CNMR of **1-1** (C_2CH_3)

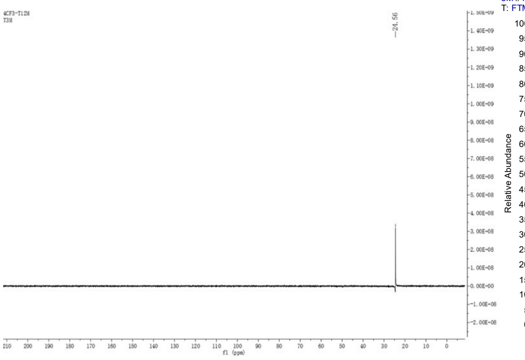

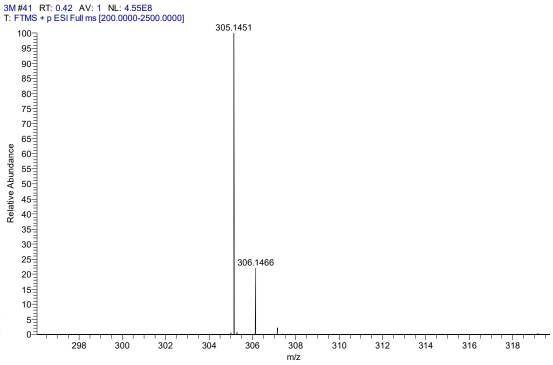

^{31}PNMR of **1-1** (C_2CH_3)　　HRMS of **1-1** (C_2CH_3)

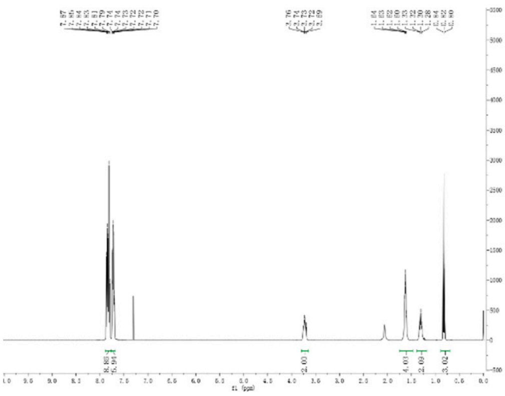

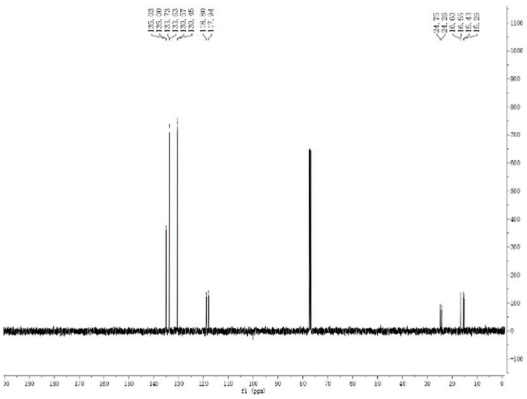

^{1}HNMR of **1-2** (C_4CH_3)　　^{13}CNMR of **1-2** (C_4CH_3)

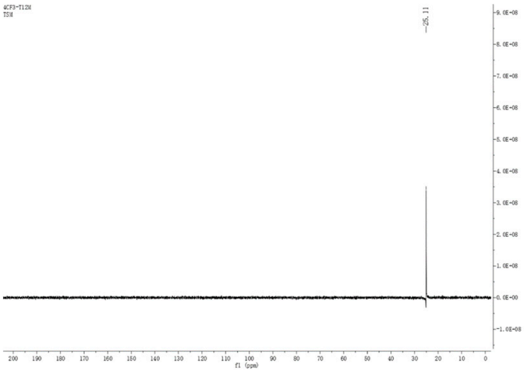

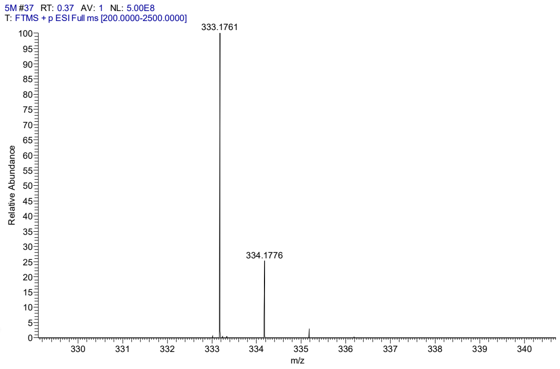

^{31}PNMR of **1-2** (C_4CH_3)　　HRMS of **1-2** (C_4CH_3)

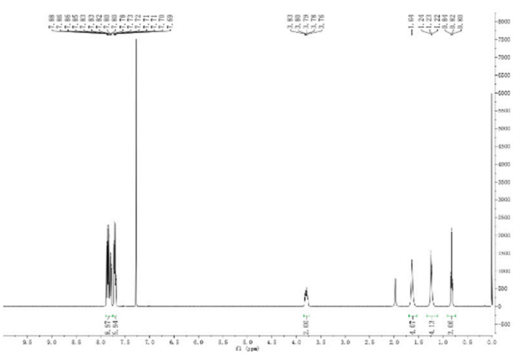

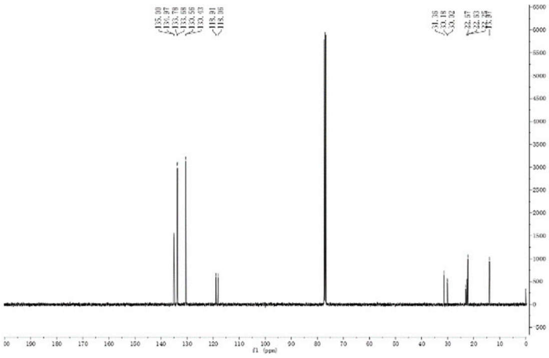

^{1}HNMR of **1-3** (C_5CH_3)　　^{13}CNMR of **1-3** (C_5CH_3)

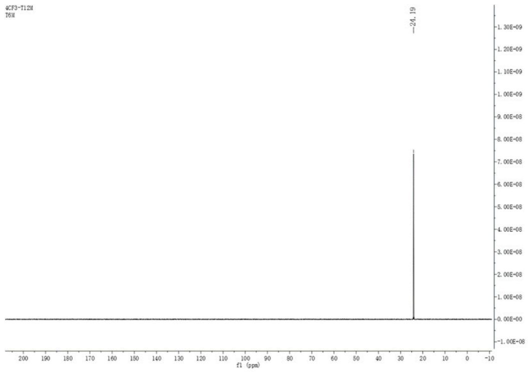

^{31}PNMR of **1-3** (C_5CH_3)

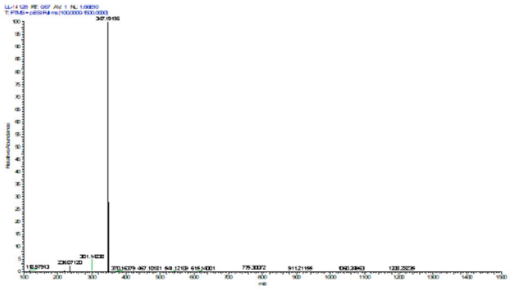

HRMS of **1-3** (C_5CH_3)

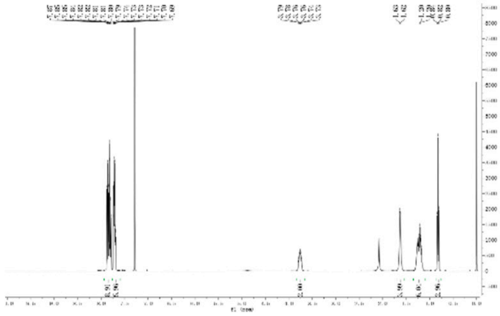

^{1}HNMR of **1-4** (C_6CH_3)

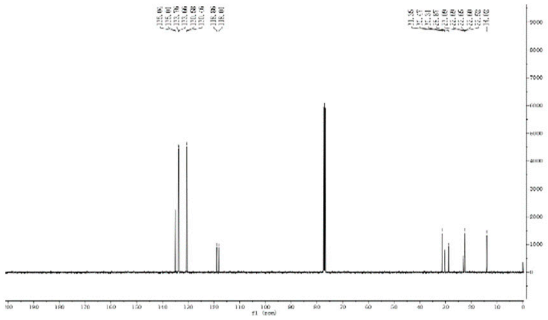

^{13}CNMR of **1-4** (C_6CH_3)

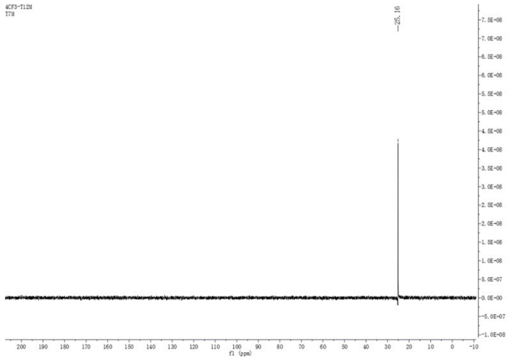

^{31}PNMR of **1-4** (C_6CH_3)

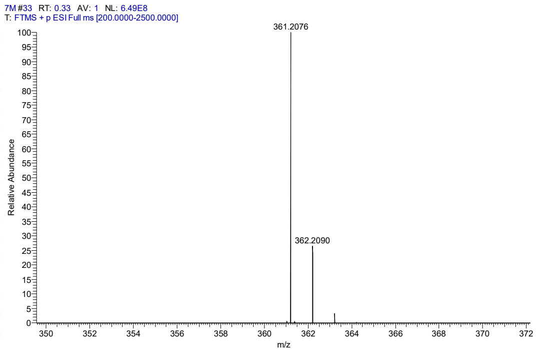

HRMS of **1-4** (C_6CH_3)

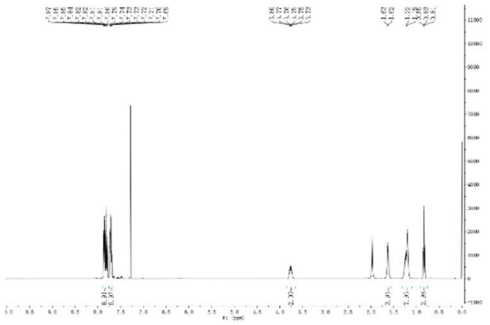

^{1}HNMR of **1-5** (C_7CH_3)

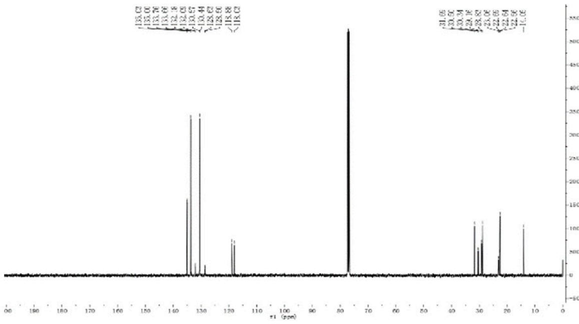

^{13}CNMR of **1-5** (C_7CH_3)

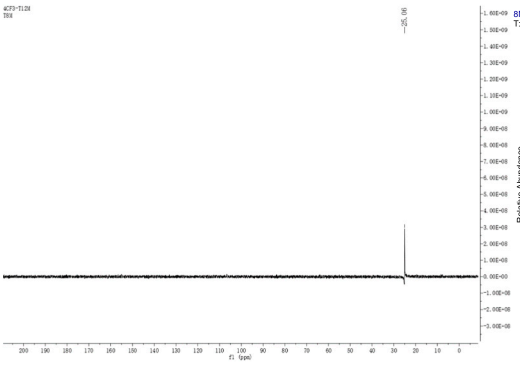

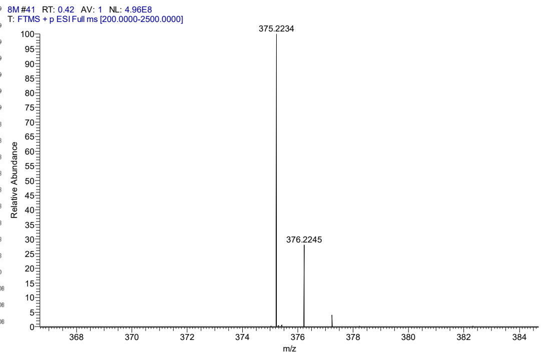

^{31}PNMR of **1-5** (C_7CH_3)

HRMS of **1-5** (C_7CH_3)

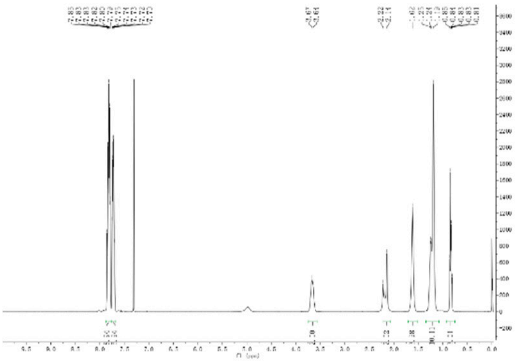

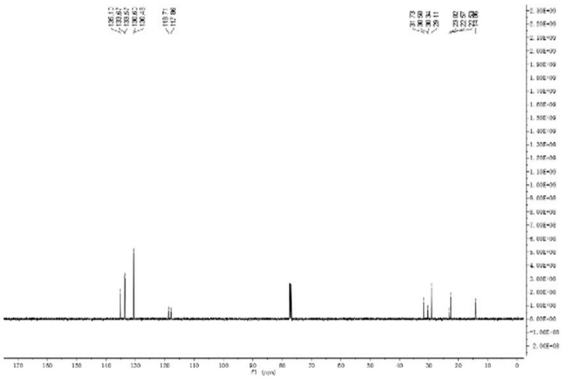

^{1}HNMR of **1-6** (C_8CH_3)

^{13}CNMR of **1-6** (C_8CH_3)

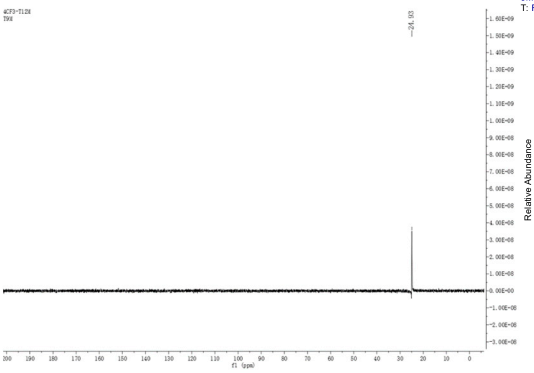

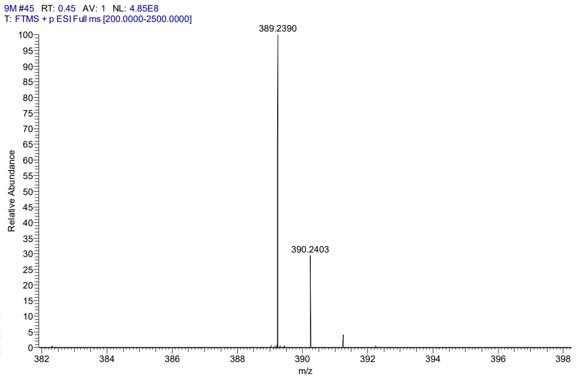

^{31}PNMR of **1-6** (C_8CH_3)

HRMS of **1-6** (C_8CH_3)

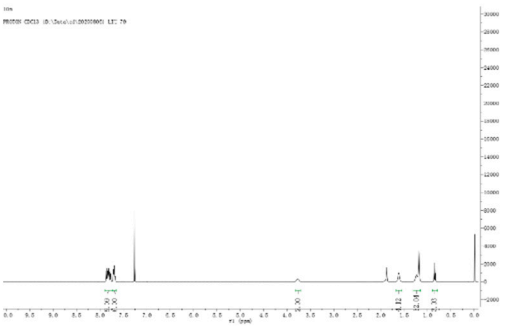

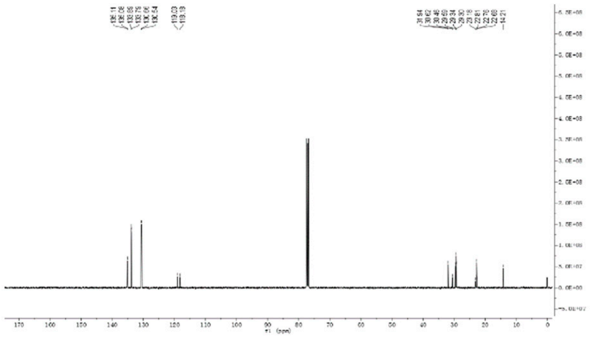

^{1}HNMR of **1-7** (C_9CH_3)

^{13}CNMR of **1-7** (C_9CH_3)

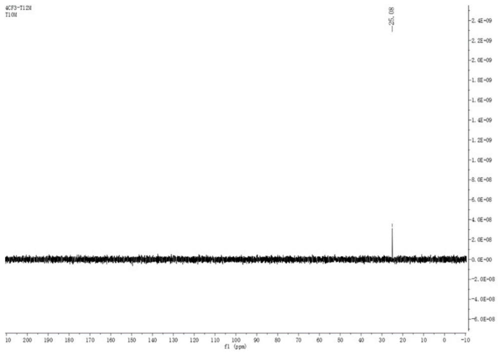

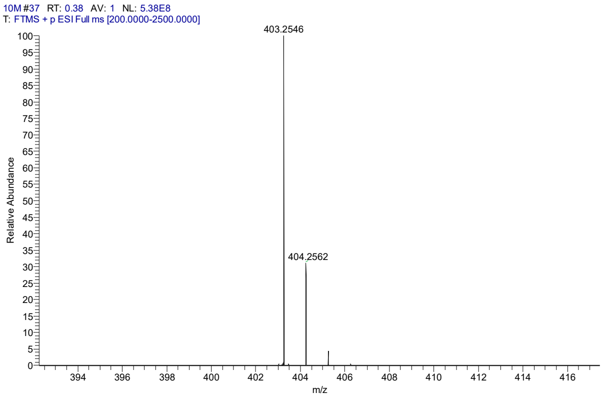

^{31}PNMR of **1-7** (C_9CH_3)

HRMS of **1-7** (C_9CH_3)

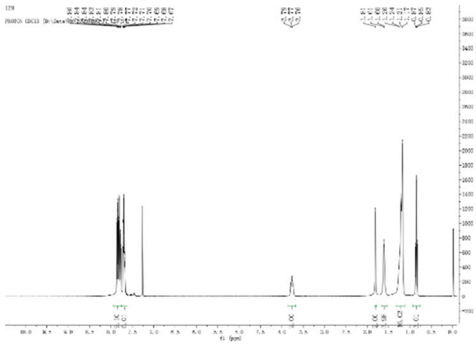

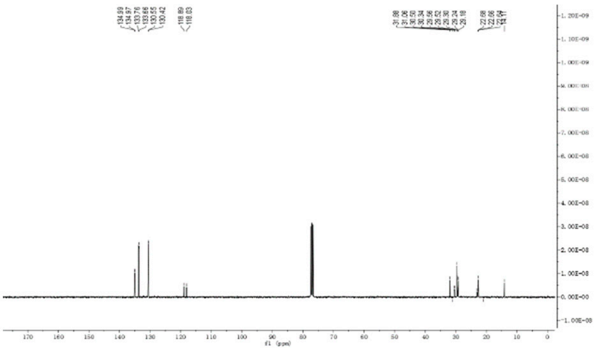

^{1}HNMR of **1-8** ($C_{11}CH_3$)

^{13}CNMR of **1-8** ($C_{11}CH_3$)

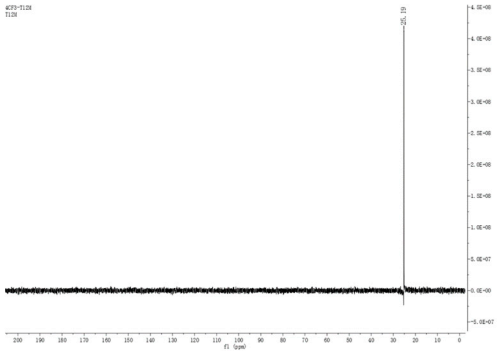

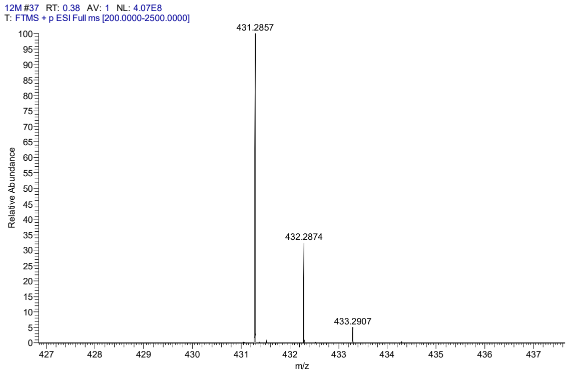

^{31}PNMR of **1-8** ($C_{11}CH_3$)

HRMS of **1-8** ($C_{11}CH_3$)

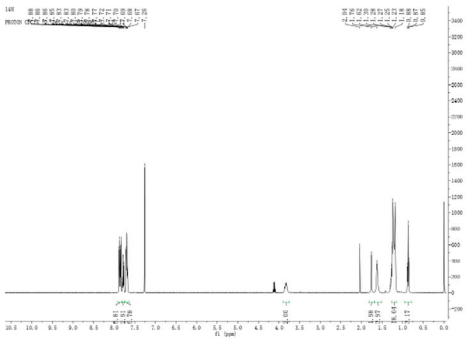

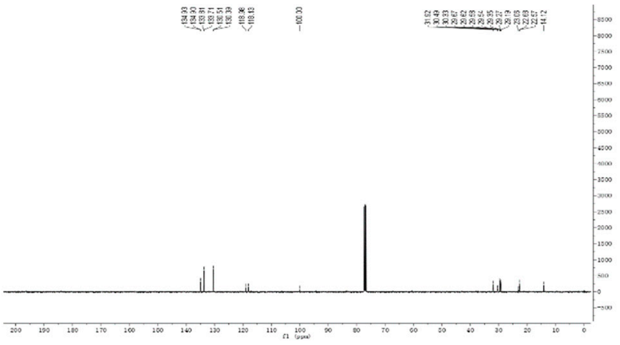

^{1}HNMR of **1-9** ($C_{13}CH_3$)

^{13}CNMR of **1-9** ($C_{13}CH_3$)

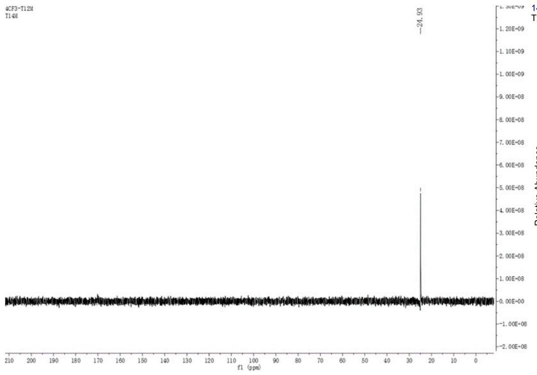

³¹PNMR of **1-9** ($C_{13}CH_3$)

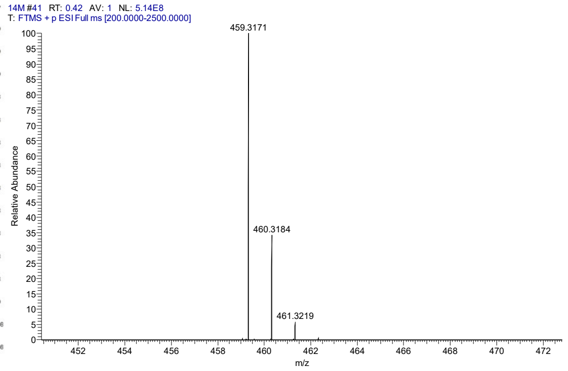

HRMS of **1-9** ($C_{13}CH_3$)

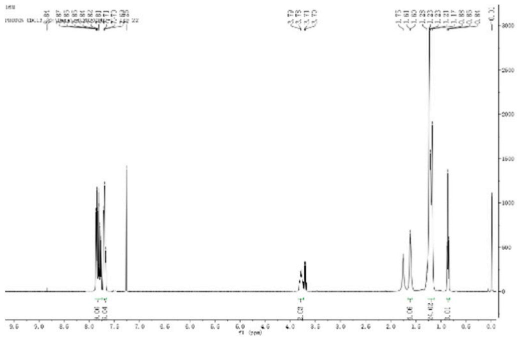

¹HNMR of **1-10** ($C_{15}CH_3$)

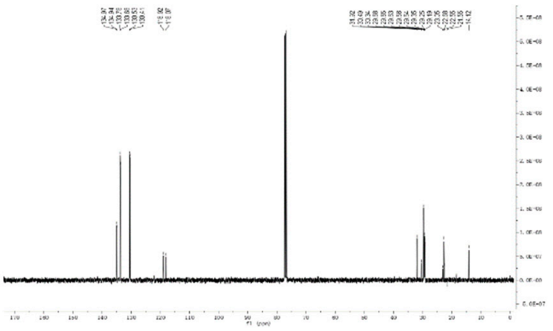

¹³CNMR of **1-10** ($C_{15}CH_3$)

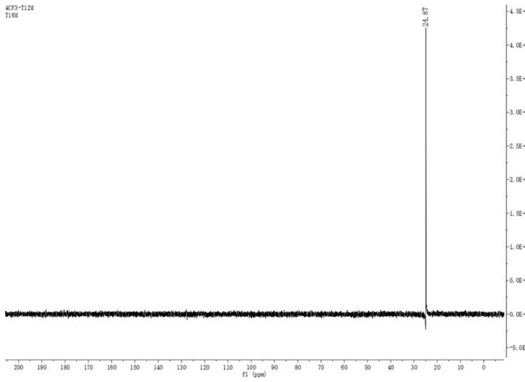

³¹PNMR of **1-10** ($C_{15}CH_3$)

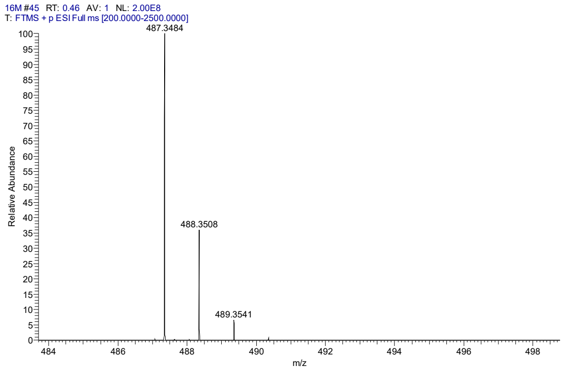

HRMS of **1-10** ($C_{15}CH_3$)

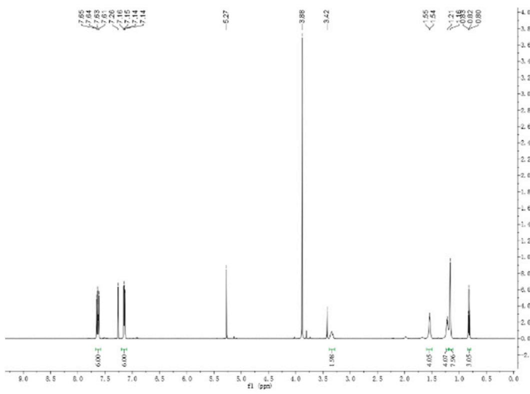

¹HNMR of **1A-1**

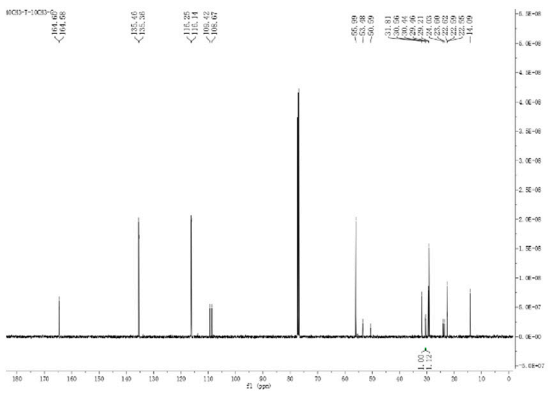

¹³CNMR of **1A-1**

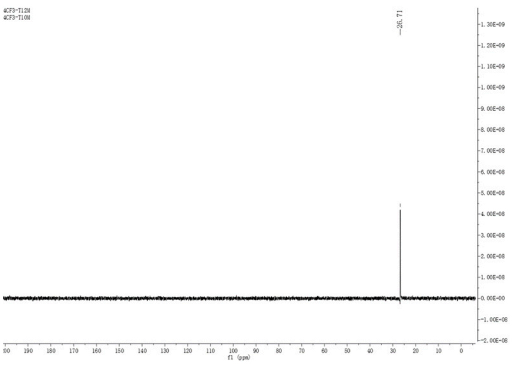

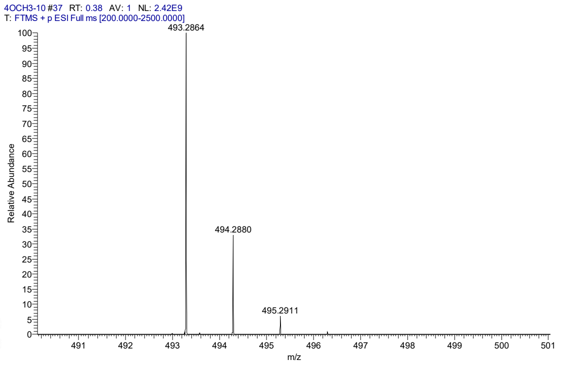

^{31}PNMR of **1A-1**

HRMS of **1A-1**

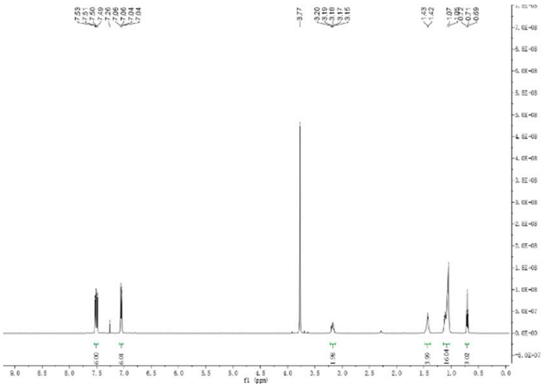

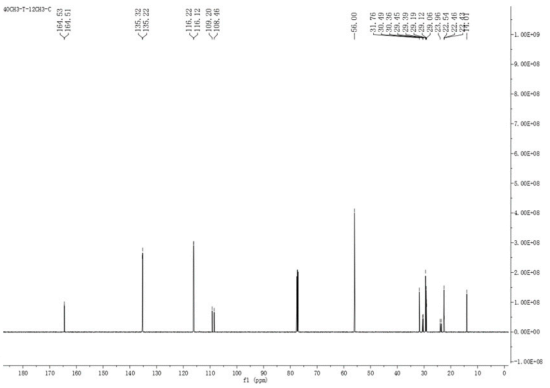

^{1}HNMR of **1A-2**

^{13}CNMR of **1A-2**

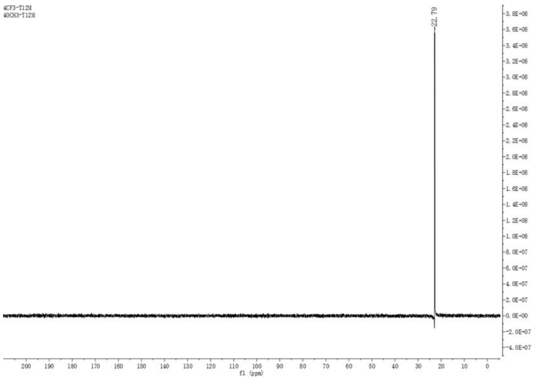

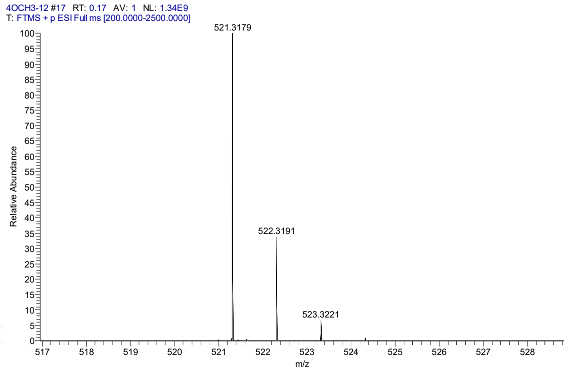

^{31}PNMR of **1A-2**

HRMS of **1A-2**

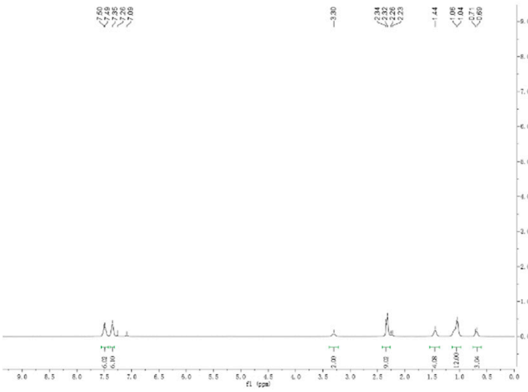

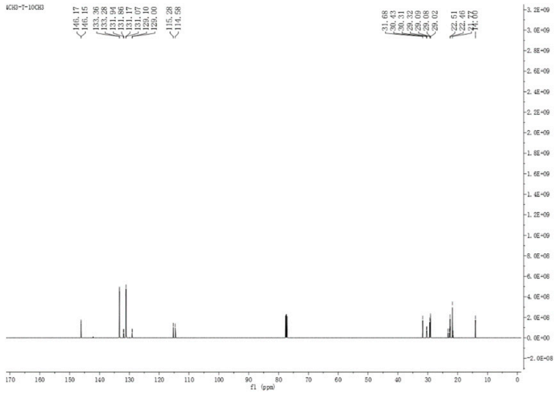

^{1}HNMR of **1B-1**

^{13}CNMR of **1B-1**

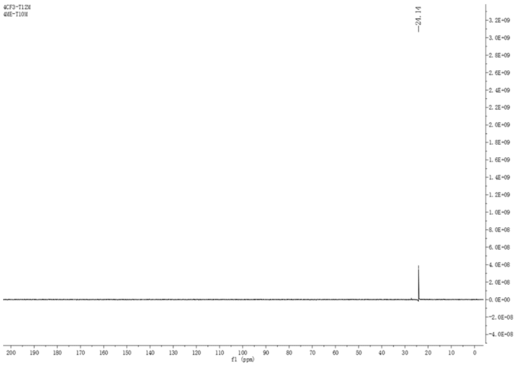

^{31}PNMR of **1B-1**

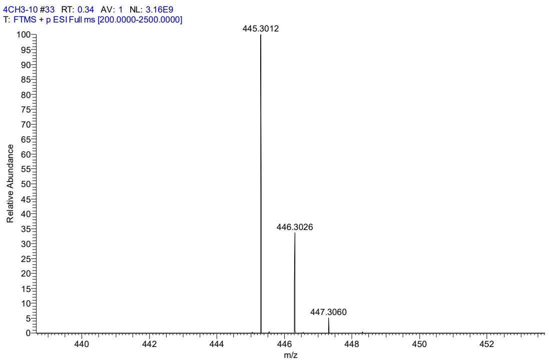

HRMS of **1B-1**

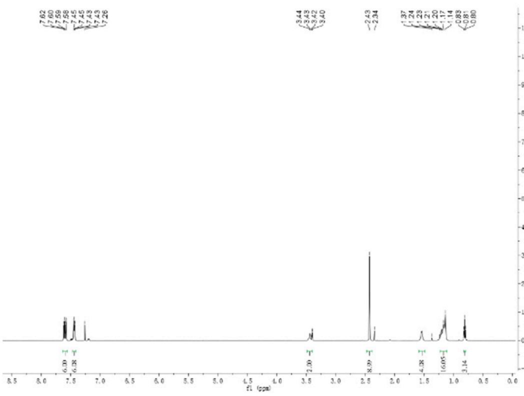

^{1}HNMR of **1B-2**

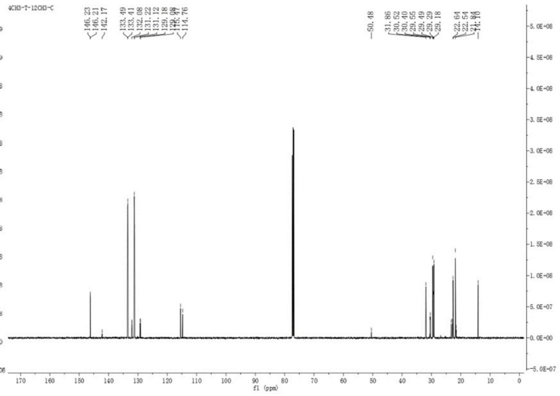

^{13}CNMR of **1B-2**

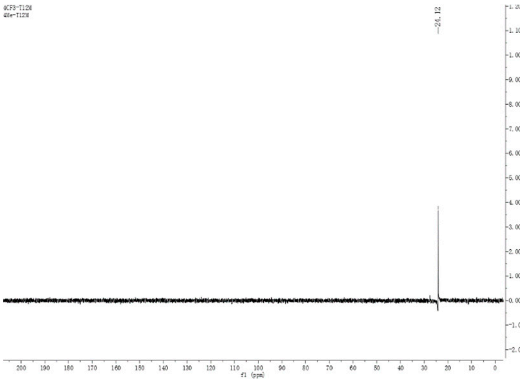

^{31}PNMR of **1B-2**

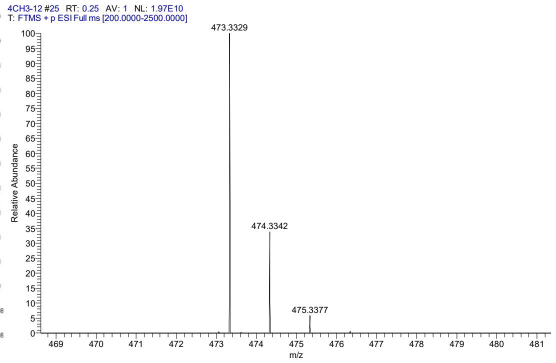

HRMS of **1B-2**

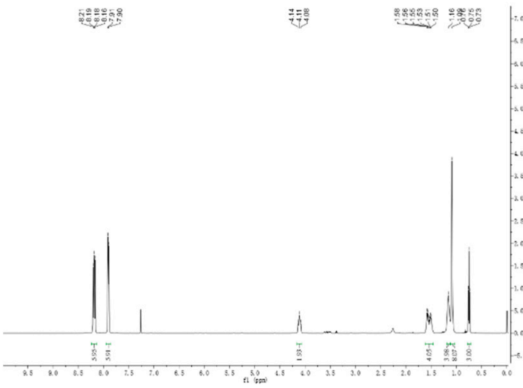

^{1}HNMR of **1C-1**

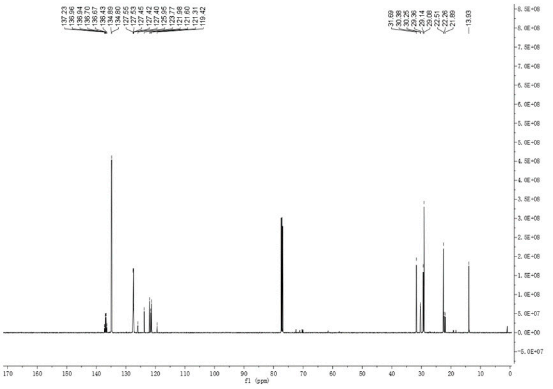

^{13}CNMR of **1C-1**

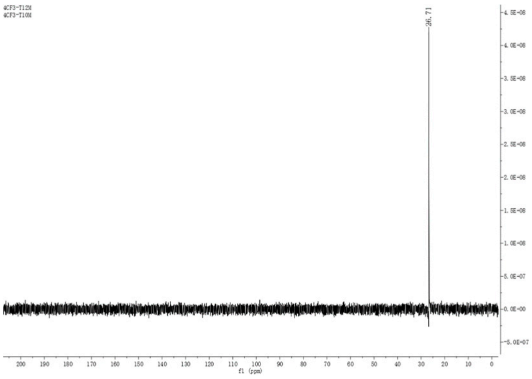

^{31}PNMR of **1C-1**

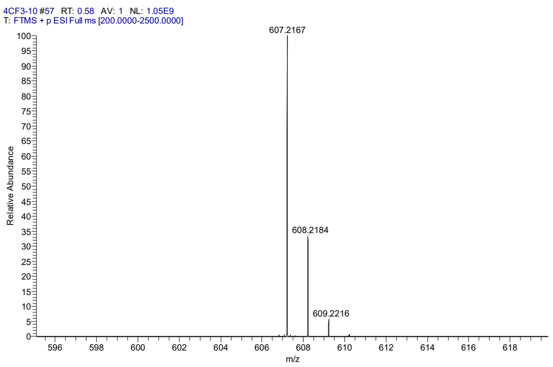

HRMS of **1C-1**

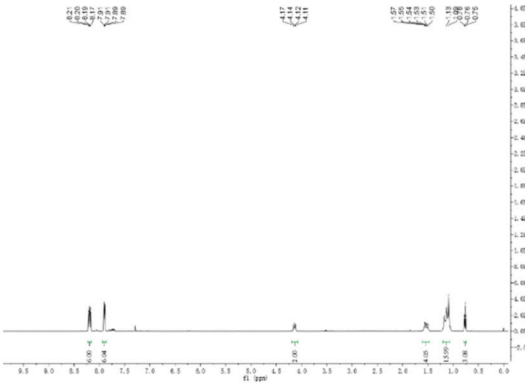

^{1}HNMR of **1C- 2**

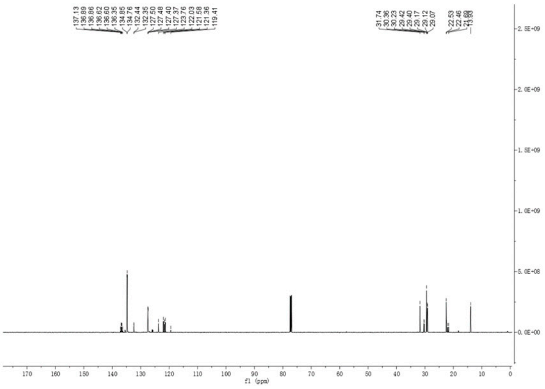

^{13}CNMR of **1C-2**

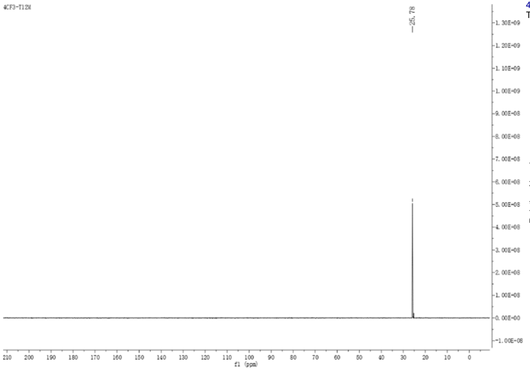

^{31}PNMR of **1C-2**

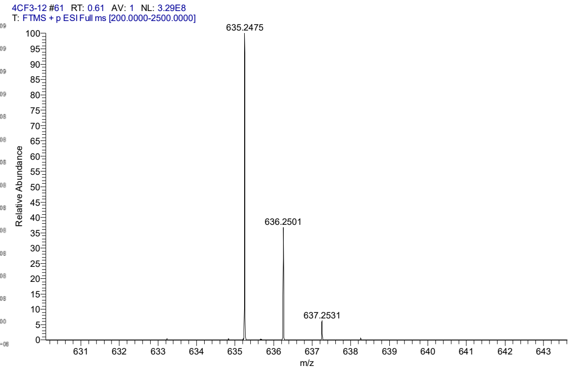

HRMS of **1C-2**

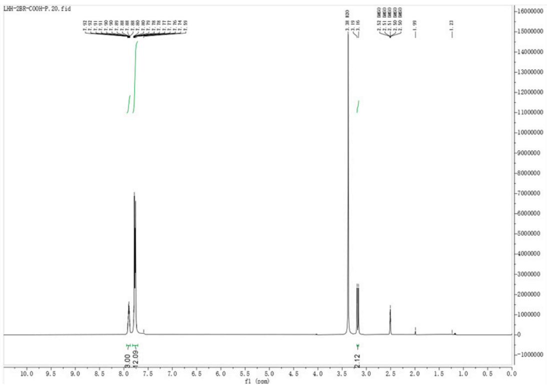

^{1}HNMR of **2-1** (C_1COOH)

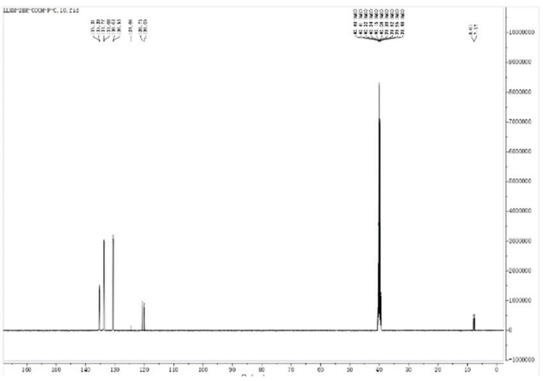

^{13}CNMR of **2-1** (C_1COOH)

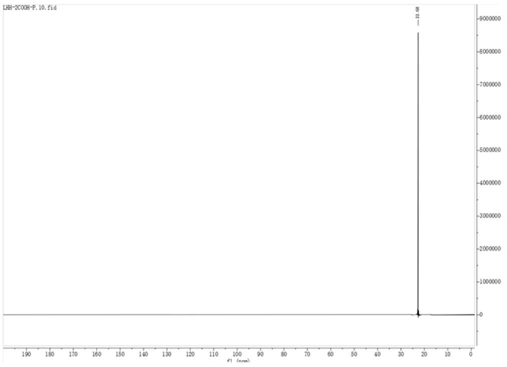

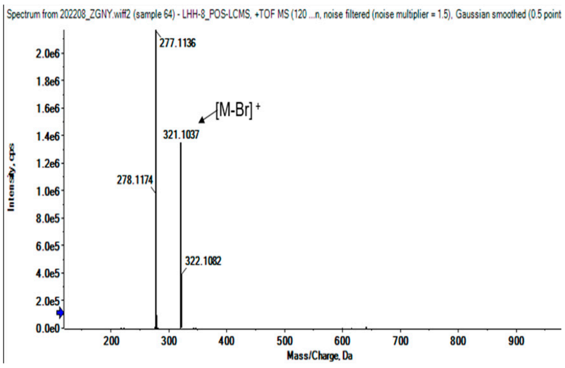

^{31}PNMR of **2-1** (C_1COOH) HRMS of **2-1** (C_1COOH)

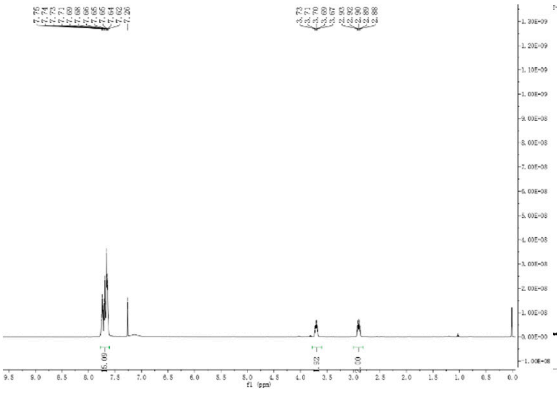

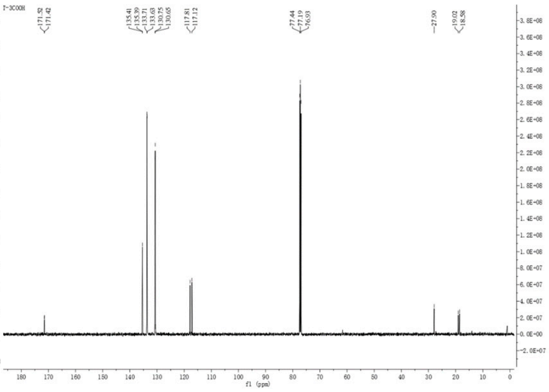

^{1}HNMR of **2-2** (C_2COOH) ^{13}CNMR of **2-2** (C_2COOH)

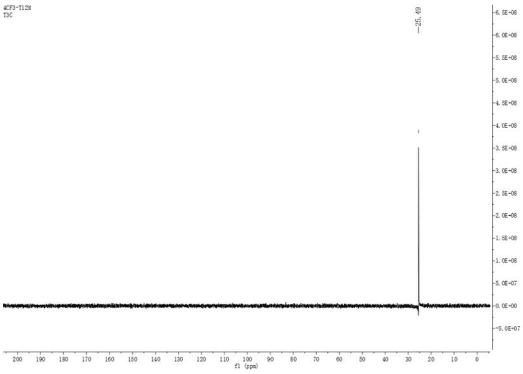

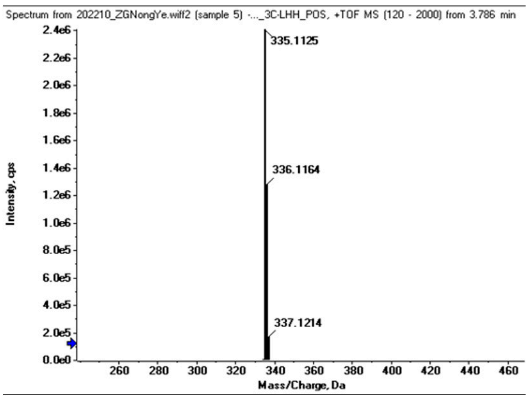

^{31}PNMR of **2-2** (C_2COOH) HRMS of **2-2** (C_2COOH)

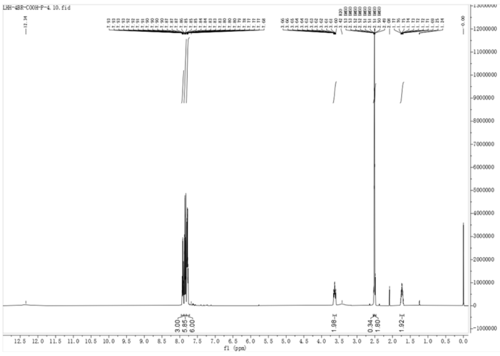

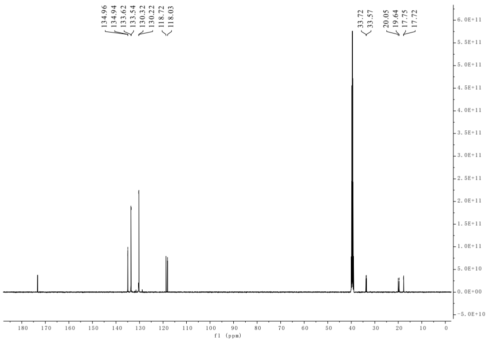

^{1}HNMR of **2-3** (C_3COOH) ^{13}CNMR of **2-3** (C_3COOH)

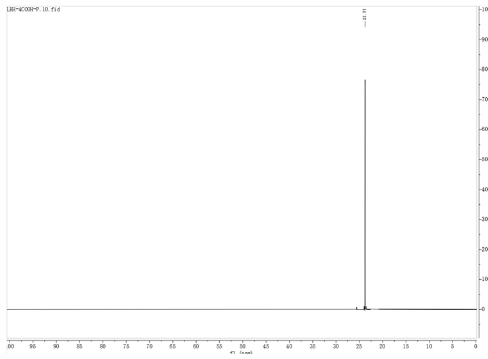

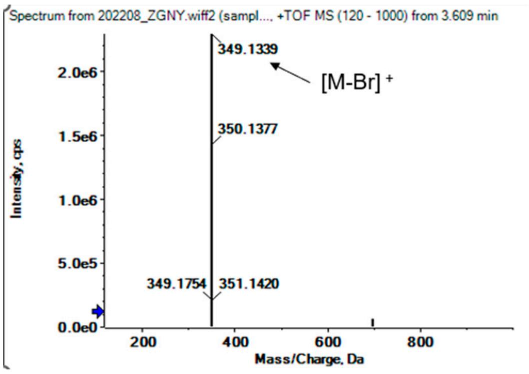

^{31}PNMR of **2-3** (C_3COOH)

HRMS of **2-3** (C_3COOH)

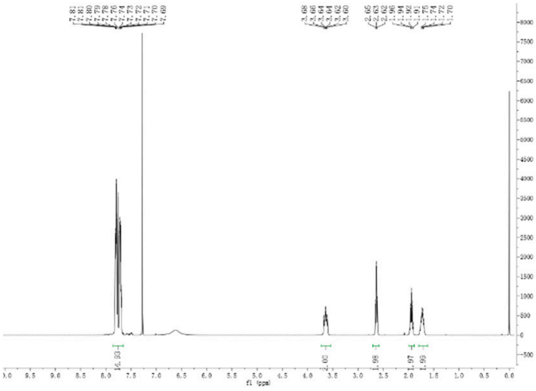

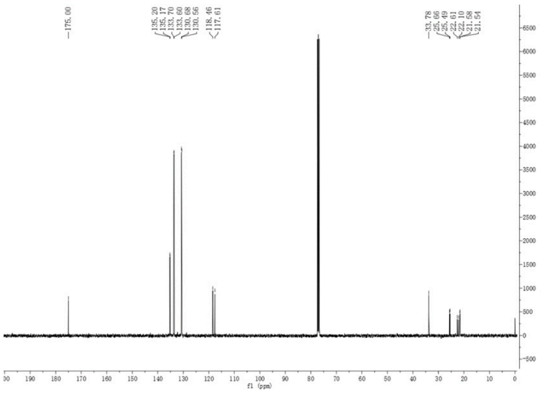

^{1}HNMR of **2-4** (C_4COOH)

^{13}CNMR of **2-4** (C_4COOH)

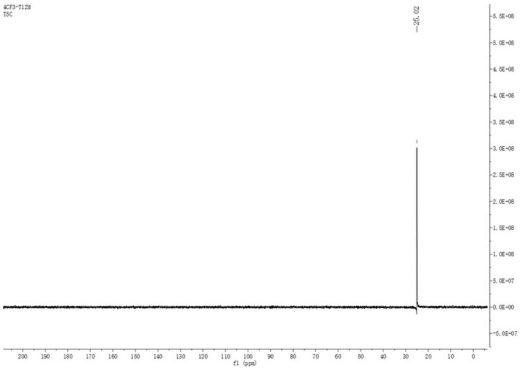

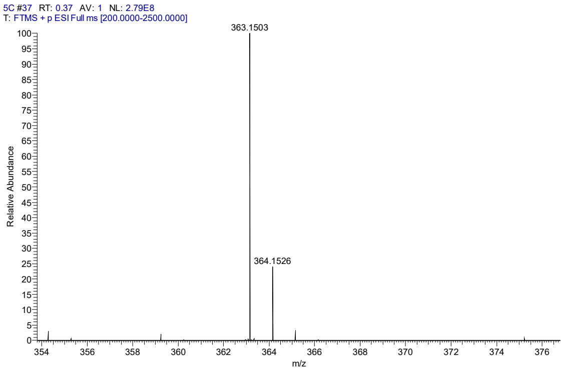

^{31}PNMR of **2-4** (C_4COOH)

HRMS of **2-4** (C_4COOH)

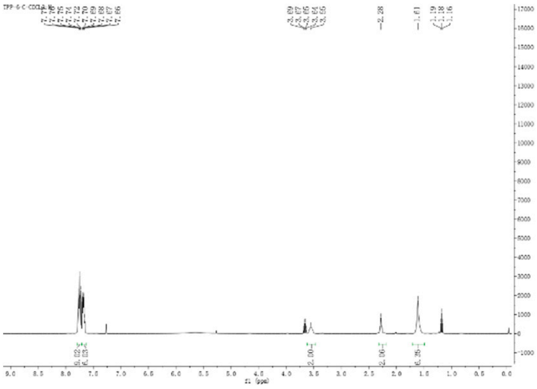

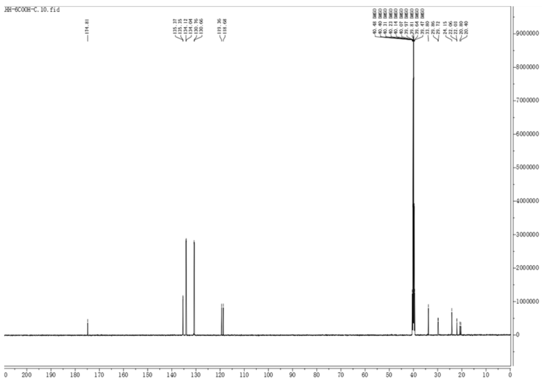

^{1}HNMR of **2-5** (C_5COOH)

^{13}CNMR of **2-5** (C_5COOH)

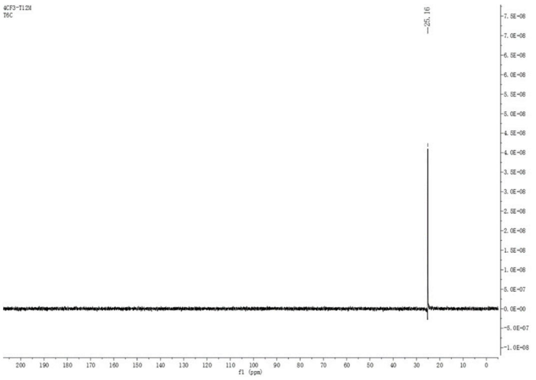

^{31}PNMR of **2-5** (C_5COOH)

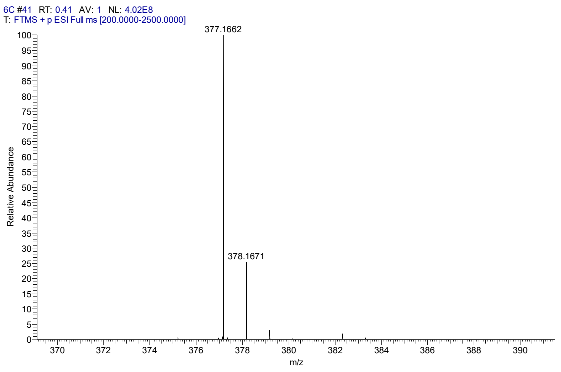

HRMS of **2-5** (C_5COOH)

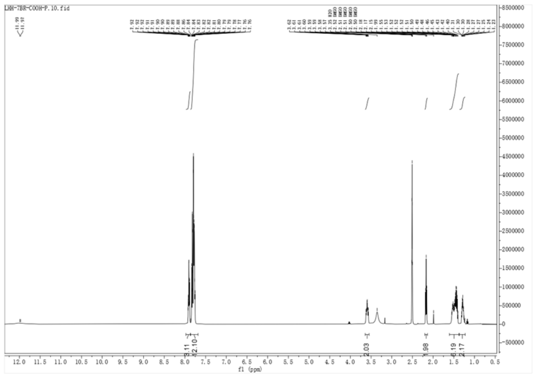

^{1}HNMR of **2-6** (C_6COOH)

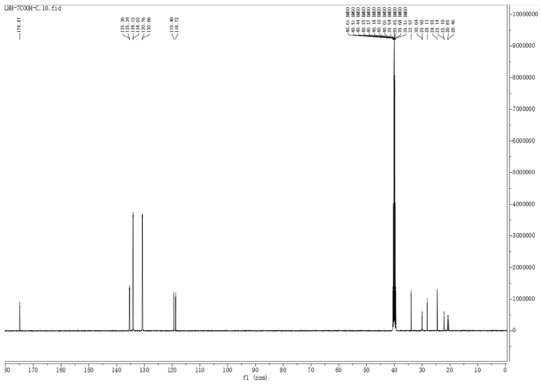

^{13}CNMR of **2-6** (C_6COOH)

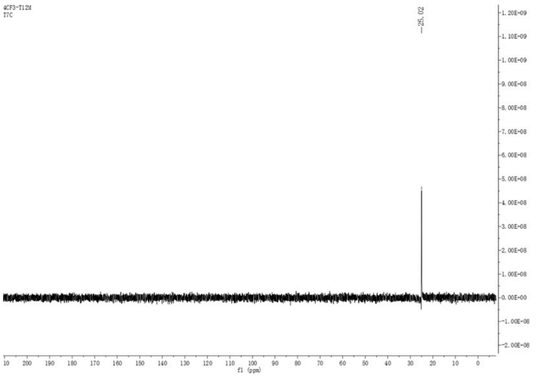

^{31}PNMR of **2-6** (C_6COOH)

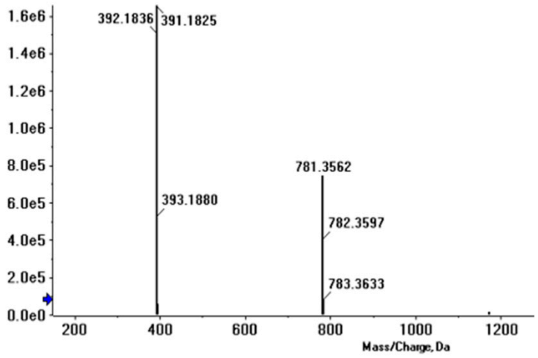

HRMS of **2-6** (C_6COOH)

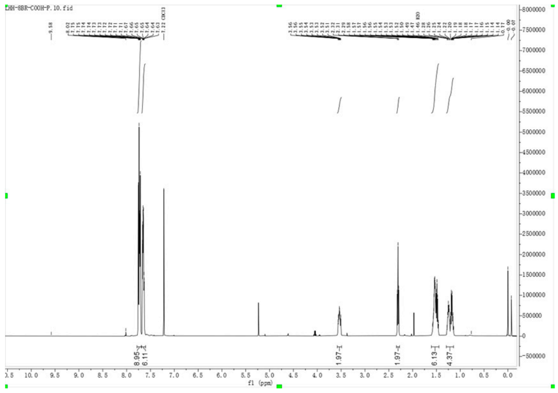

^{1}HNMR of **2-7** (C_7COOH)

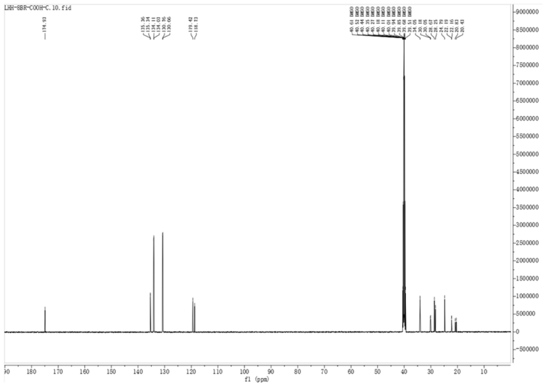

^{13}CNMR of **2-7** (C_7COOH)

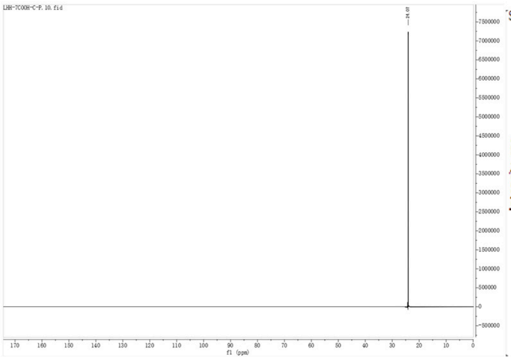
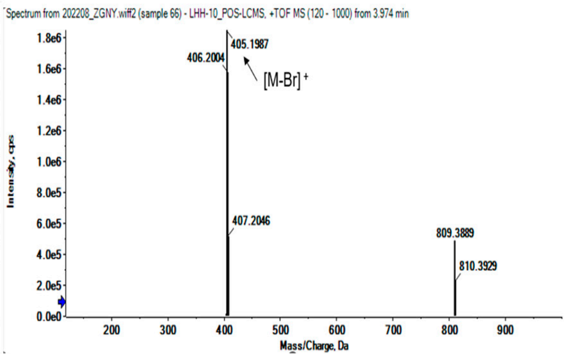

^{31}PNMR of **2-7** (C_7COOH)

HRMS of **2-7** (C_7COOH)

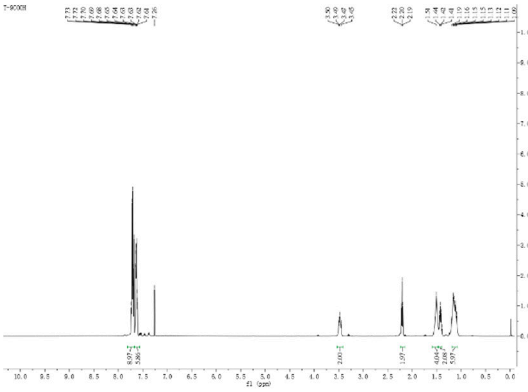
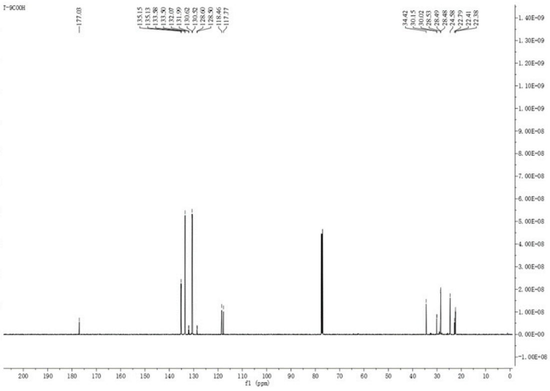

^{1}HNMR of **2-8** (C_8COOH)

^{13}CNMR of **2-8** (C_8COOH)

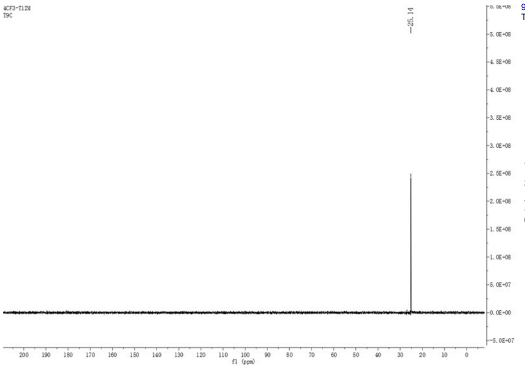
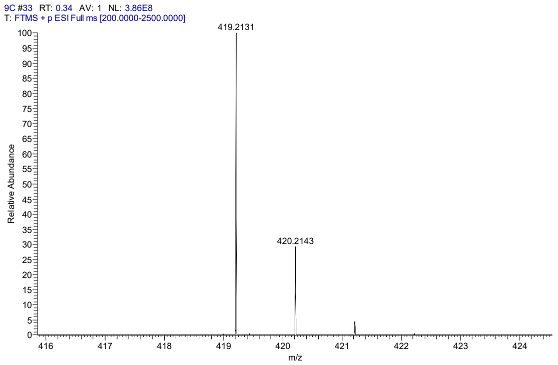

^{31}PNMR of **2-8** (C_8COOH)

HRMS of **2-8** (C_8COOH)

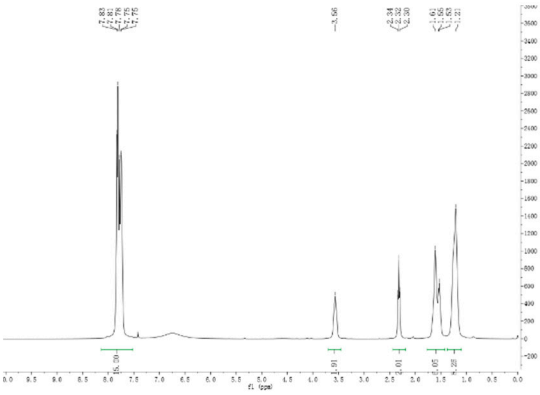
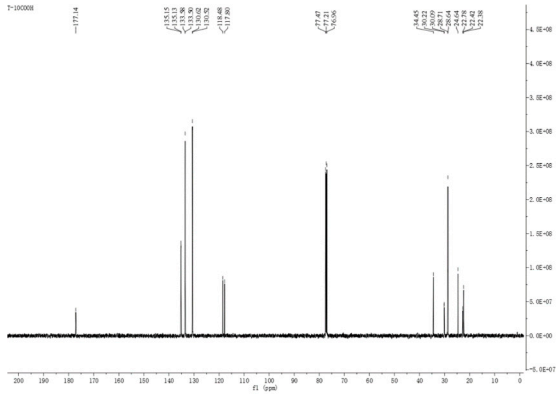

^{1}HNMR of **2-9** (C_9COOH)

^{13}CNMR of **2-9** (C_9COOH)

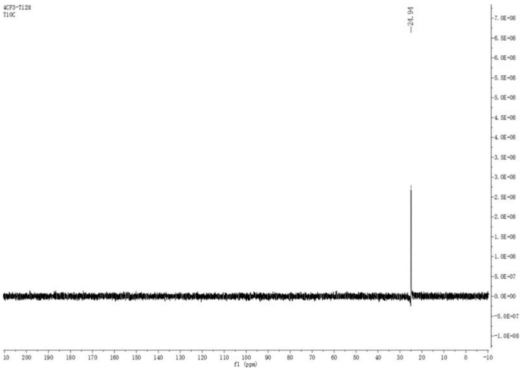

^{31}PNMR of **2-9** (C_9COOH)

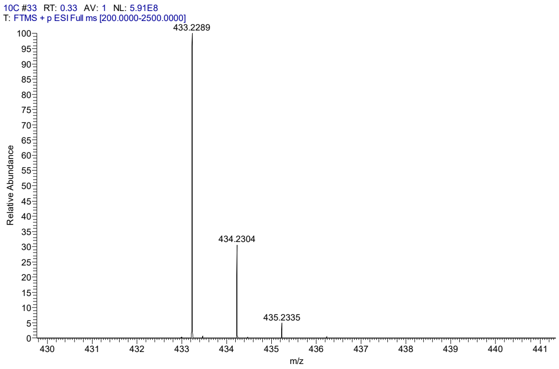

HRMS of **2-9** (C_9COOH)

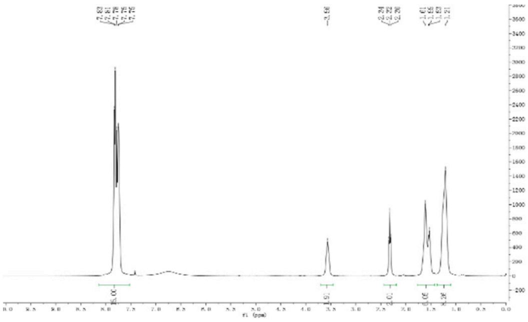

^{1}HNMR of **2-10**($C_{10}COOH$)

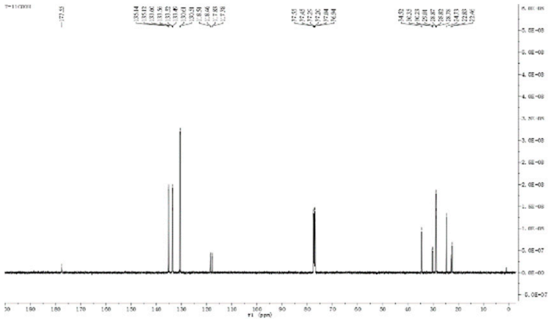

^{13}CNMR of **2-10**($C_{10}COOH$)

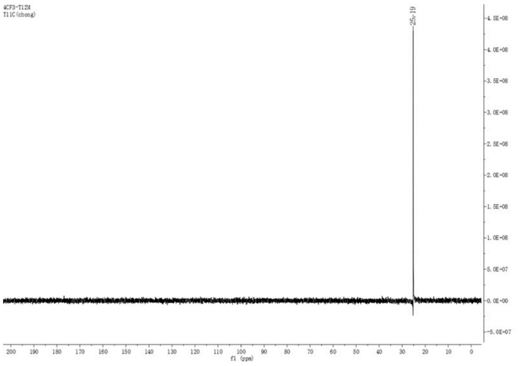

^{31}PNMR of **2-10**($C_{10}COOH$)

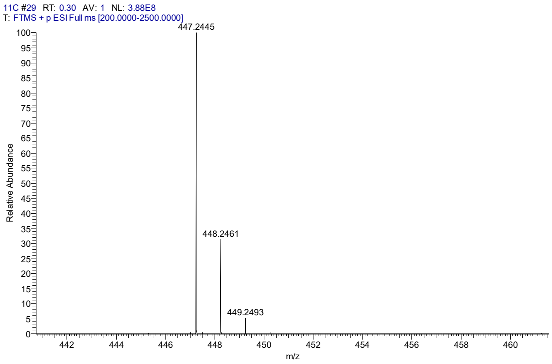

HRMS of **2-10** ($C_{10}COOH$)

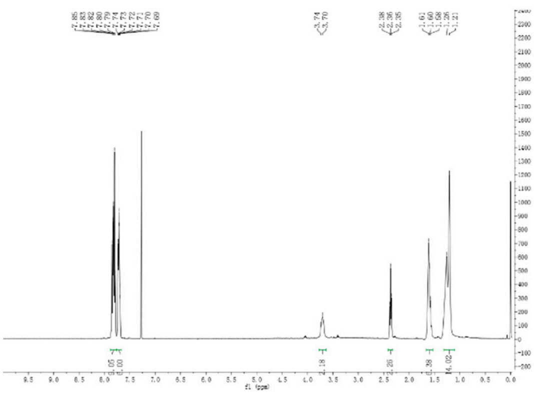

^{1}HNMR of **2-11** ($C_{11}COOH$)

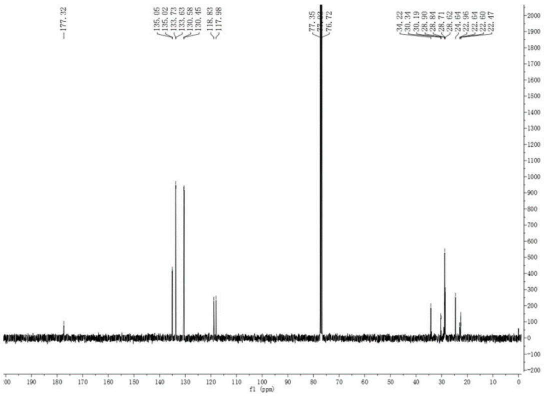

^{13}CNMR of **2-11** ($C_{11}COOH$)

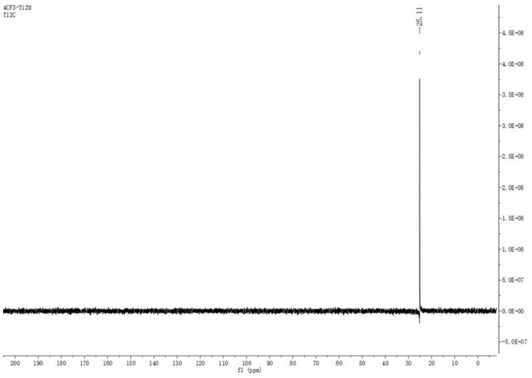

^{31}PNMR of **2-11** ($C_{11}COOH$)

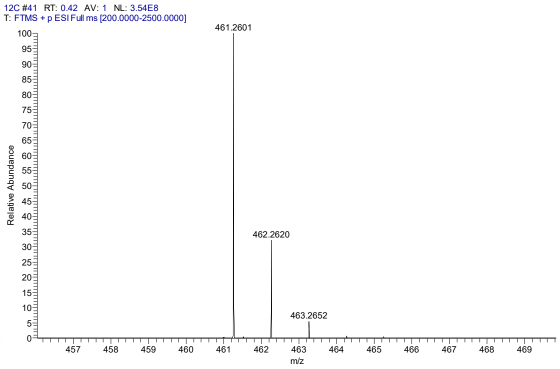

HRMS of **2-11** ($C_{11}COOH$)

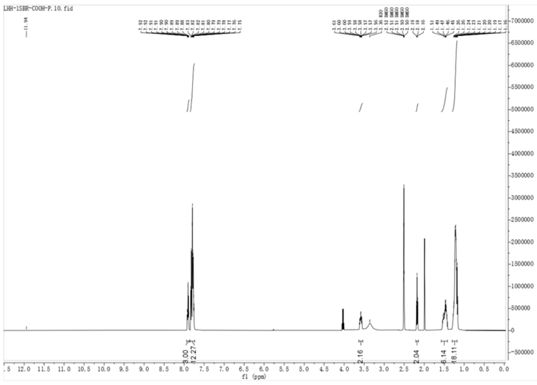

^{1}HNMR of **2-12** ($C_{14}COOH$)

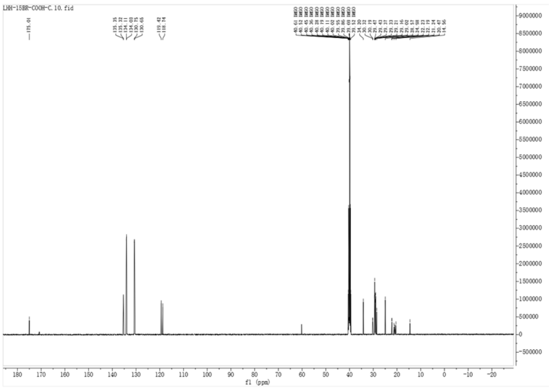

^{13}CNMR of **2-12** ($C_{14}COOH$)

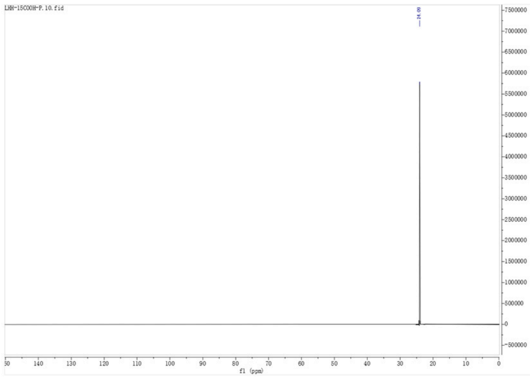

^{31}PNMR of **2-12** ($C_{14}COOH$)

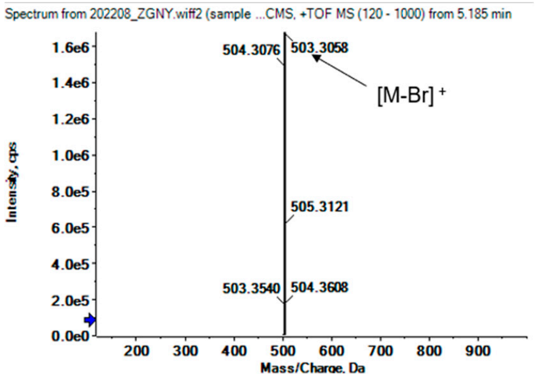

HRMS of **2-12** ($C_{14}COOH$)

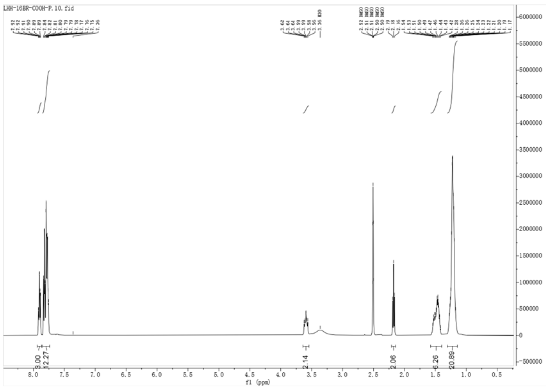

^{1}HNMR of **2-13** ($C_{15}COOH$)

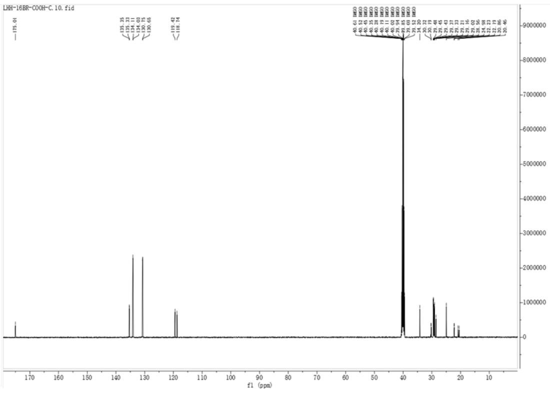

^{13}CNMR of **2-13** ($C_{15}COOH$)

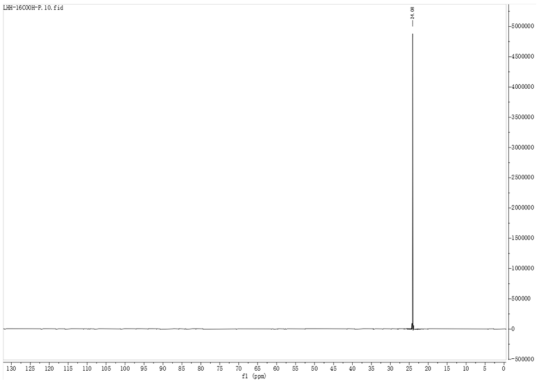

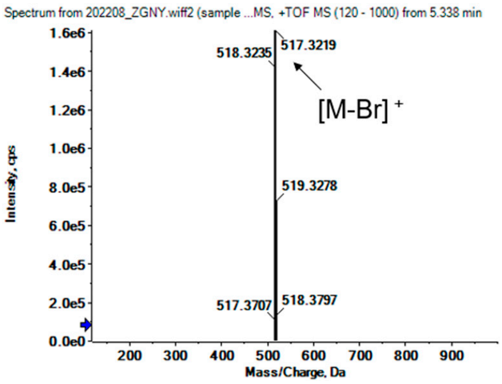

^{31}PNMR of **2-13** (C_{15}COOH)

HRMS of **2-13** (C_{15}COOH)

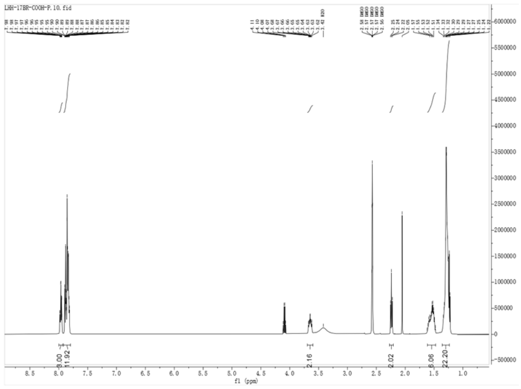

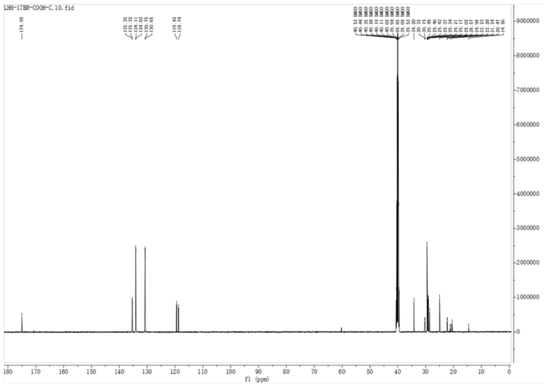

^{1}HNMR of **2-14** (C_{16}COOH)

^{13}CNMR of **2-14** (C_{16}COOH)

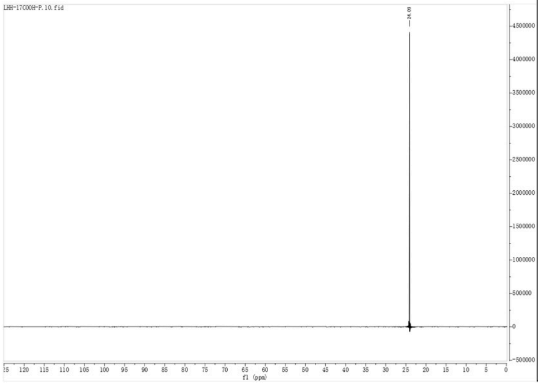

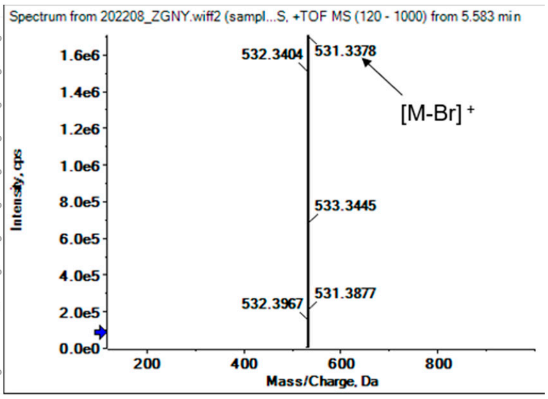

^{31}PNMR of **2-14** (C_{16}COOH)

HRMS of **2-14** (C_{16}COOH)

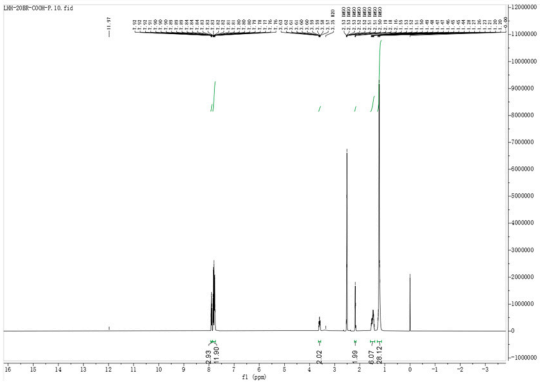

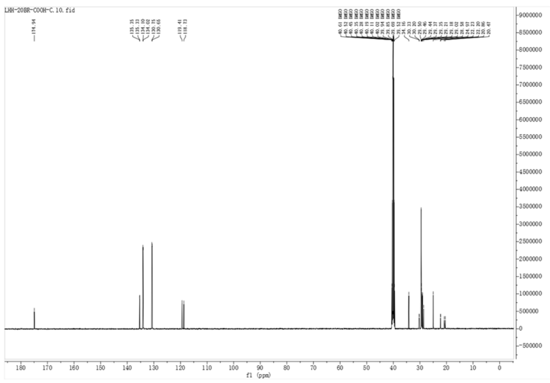

^{1}HNMR of **2-15** (C_{19}COOH)

^{13}CNMR of **2-15** (C_{19}COOH)

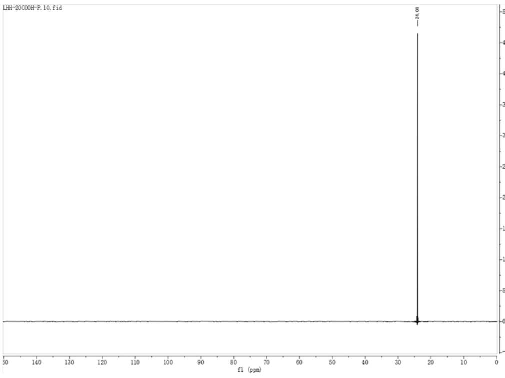

^{31}PNMR of **2-15** ($C_{19}COOH$)

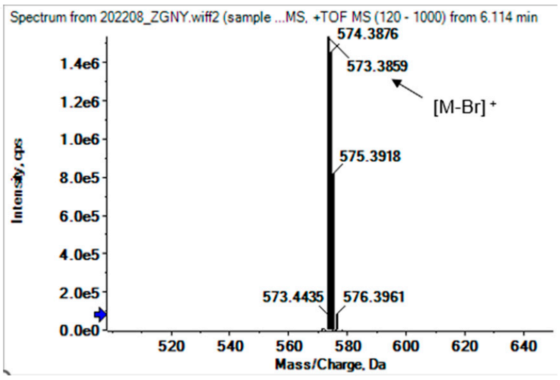

HRMS of **2-15** ($C_{19}COOH$)

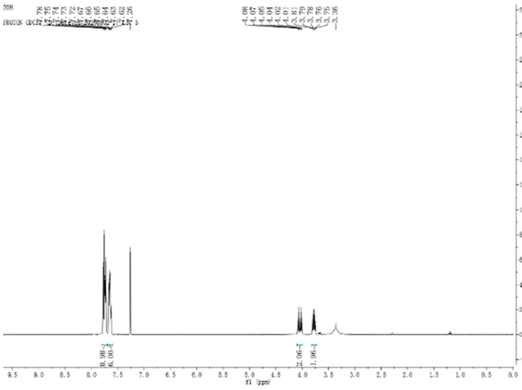

^{1}HNMR of **3-1** (C_2OH)

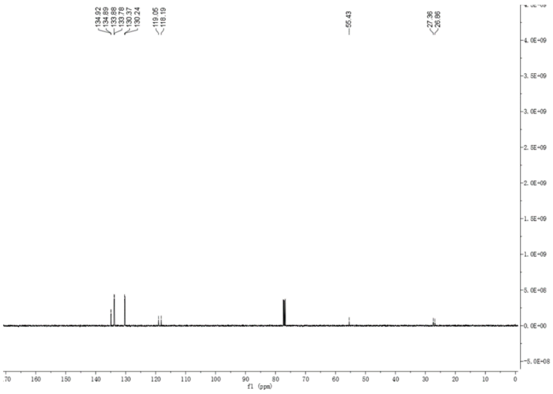

^{13}CNMR of **3-1**(C_2OH)

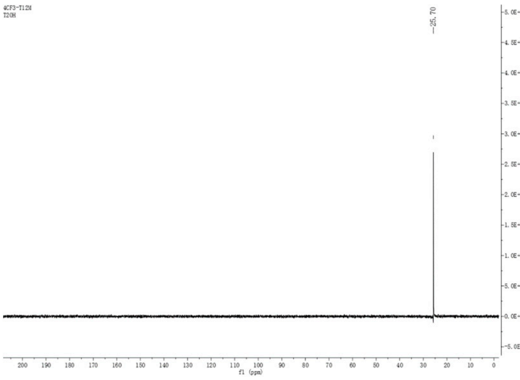

^{31}PNMR of **3-1**(C_2OH)

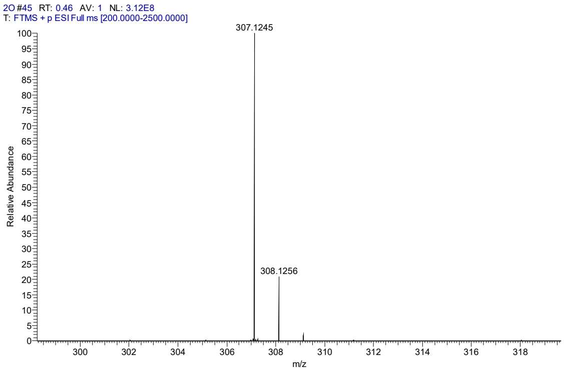

HRMS of **3-1** (C_2OH)

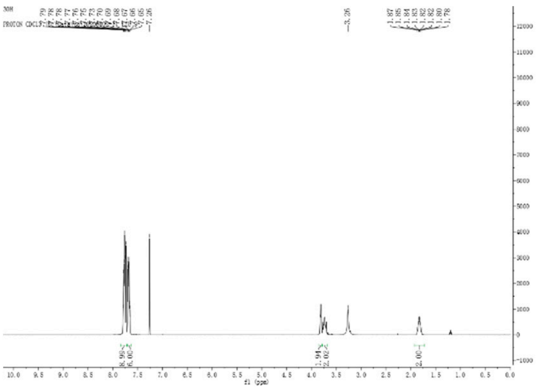

1HNMR of **3-2** (C3OH)

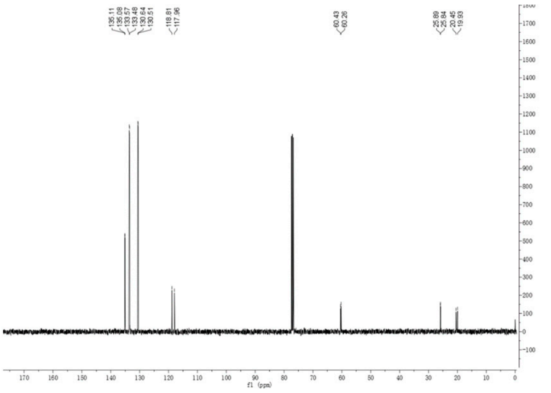

13CNMR of **3-2**(C3OH)

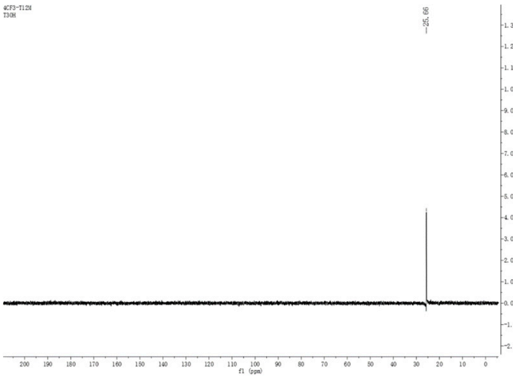

^{31}PNMR of **3-2**(C_3OH)

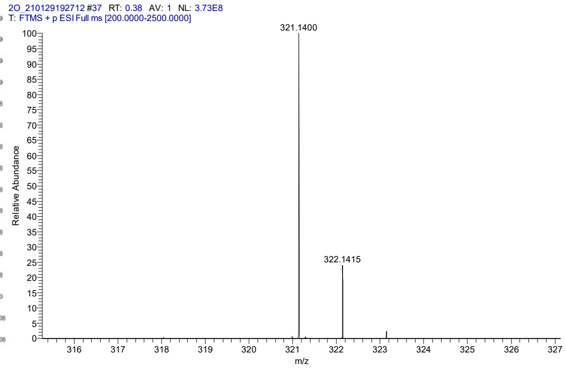

HRMS of **3-2** (C_3OH)

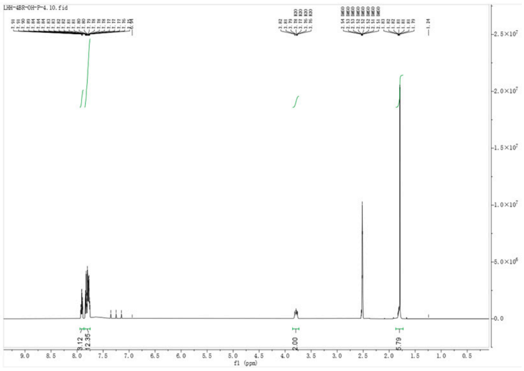

^{1}HNMR of **3-3** (C_4OH)

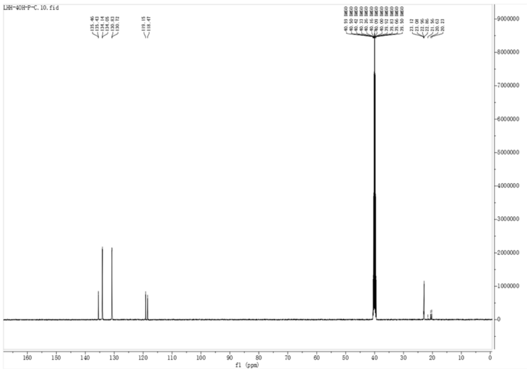

^{13}CNMR of **3-3**(C_4OH)

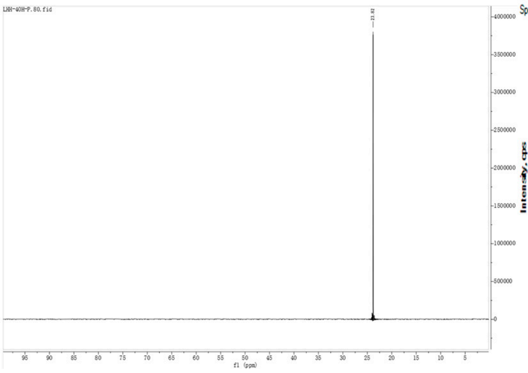

^{31}PNMR of **3-3**(C_4OH)

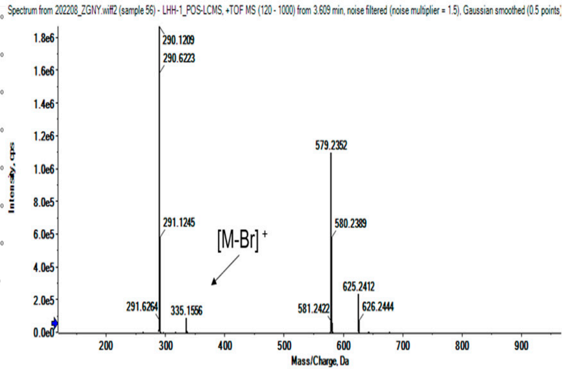

HRMS of **3-3** (C_4OH)

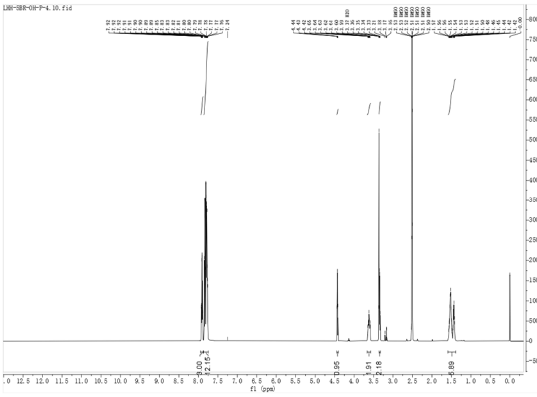

^{1}HNMR of **3-4** (C_5OH)

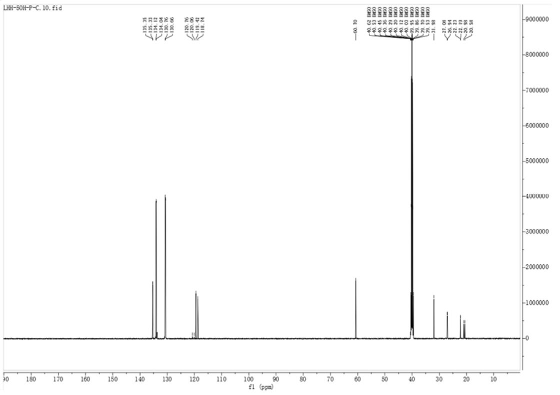

^{13}CNMR of **3-4**(C_5OH)

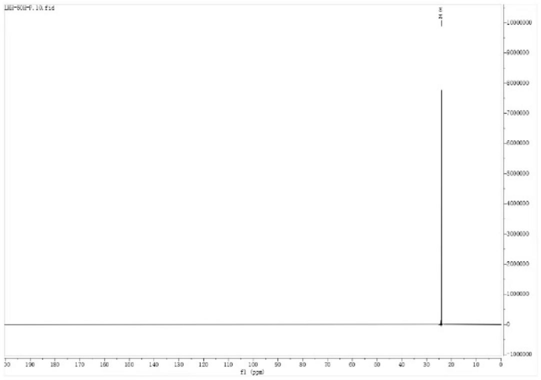

^{31}PNMR of **3-4**(C_5OH)

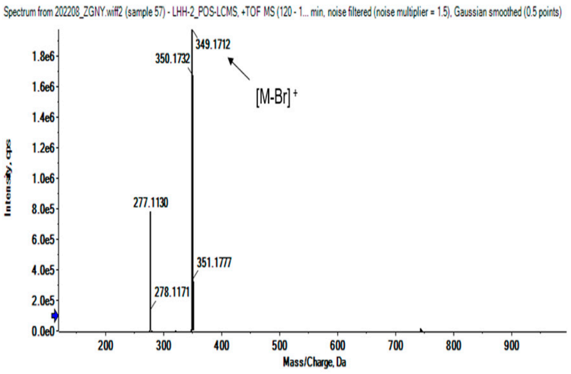

HRMS of **3-4** (C_5OH)

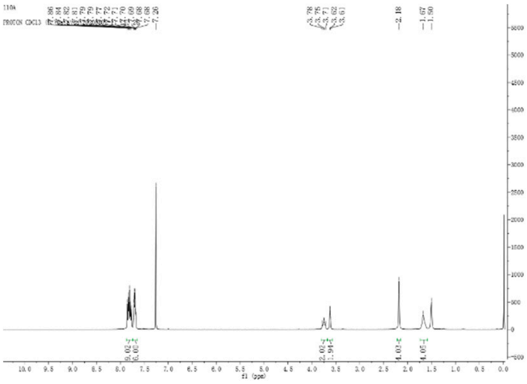

^{1}HNMR of **3-5** (C_6OH)

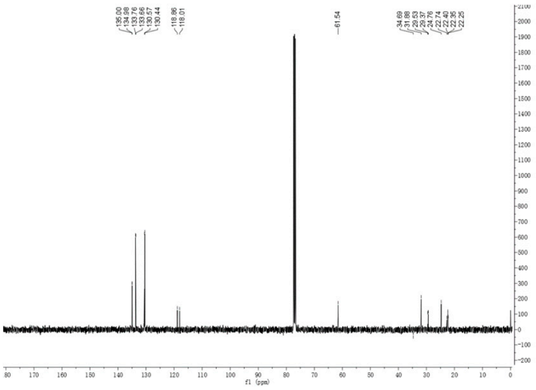

^{13}CNMR of **3-5**(C_6OH)

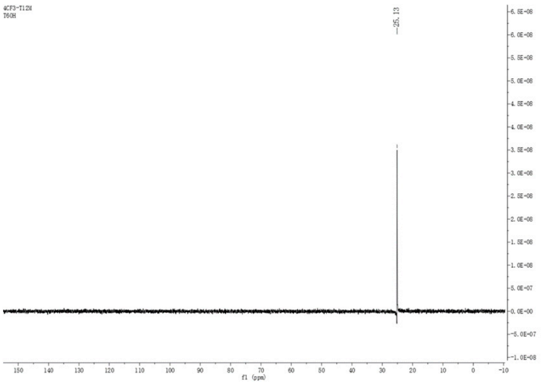

^{31}PNMR of **3-5**(C_6OH)

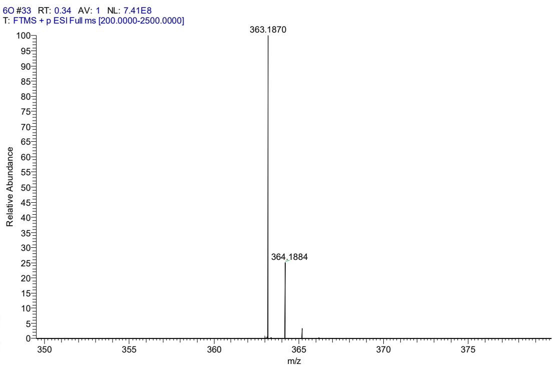

HRMS of **3-5** (C_6OH)

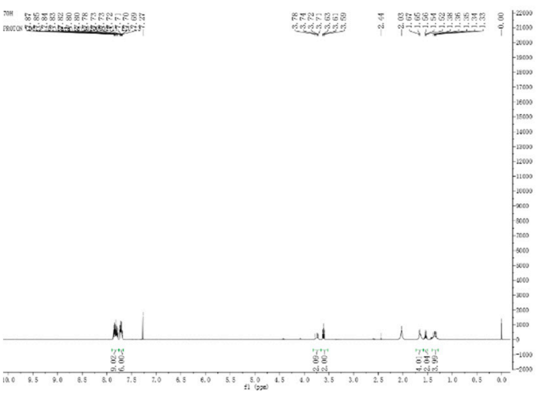

^{1}HNMR of **3-6** (C_7OH)

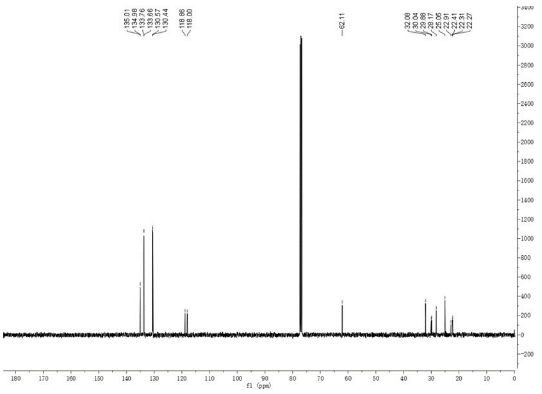

^{13}CNMR of **3-6**(C_7OH)

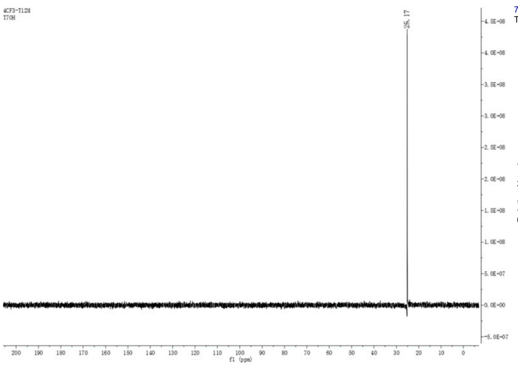

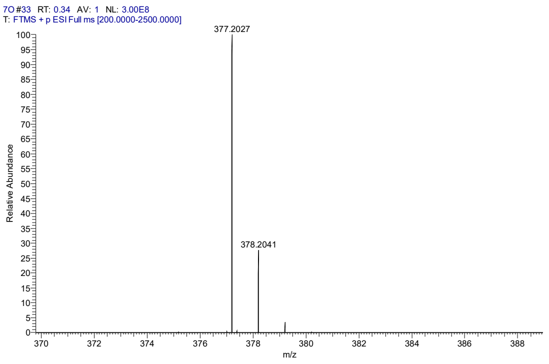

^{31}PNMR of 3-6(C_7OH) HRMS of 3-6 (C_7OH)

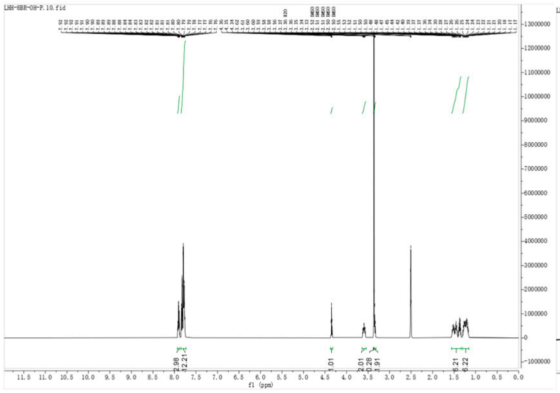

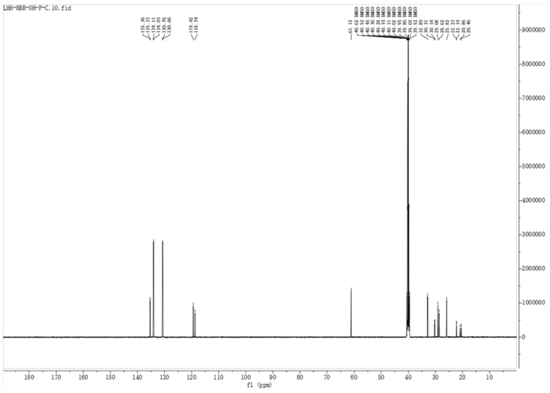

^{1}HNMR of **3-7** (C_8OH) ^{13}CNMR of **3-7**(C_8OH)

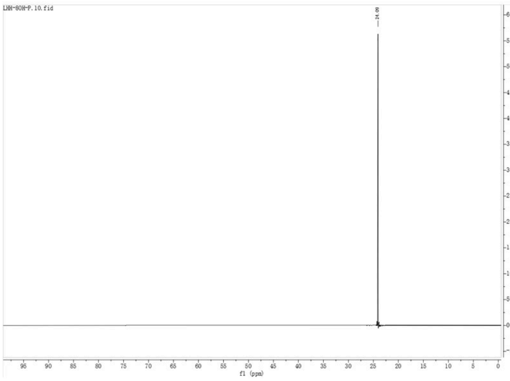

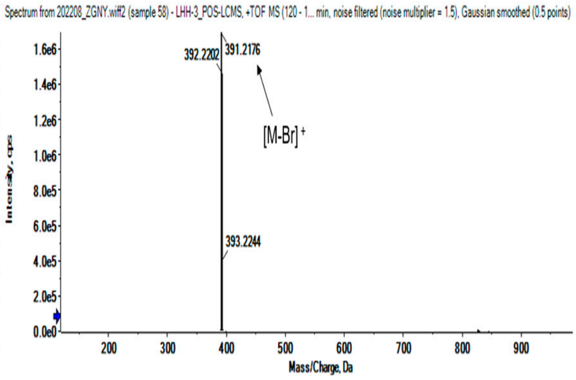

^{31}PNMR of **3-7**(C_8OH) HRMS of **3-7** (C_8OH)

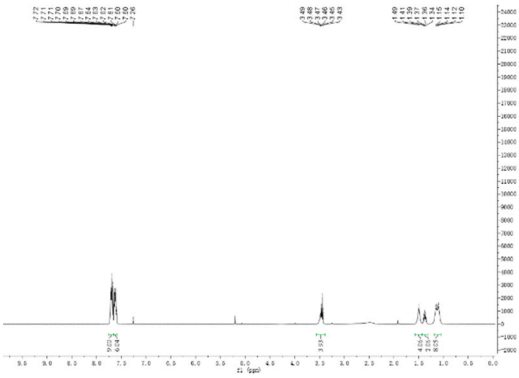

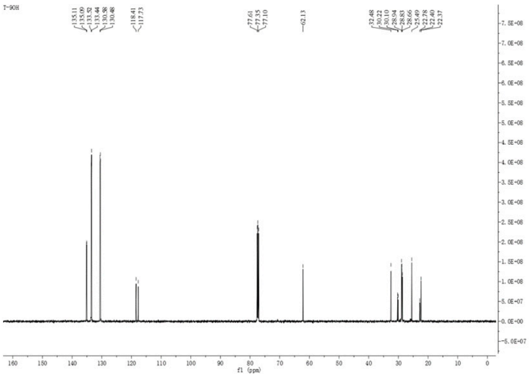

^{1}HNMR of **3-8** (C_9OH) ^{13}CNMR of **3-8**(C_9OH)

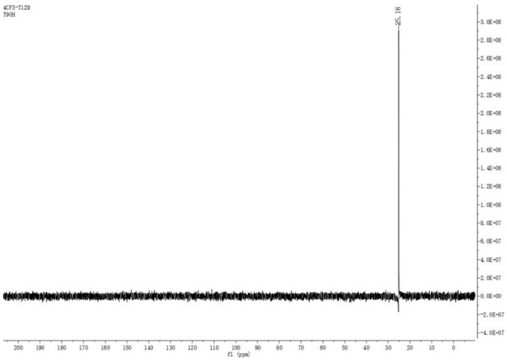

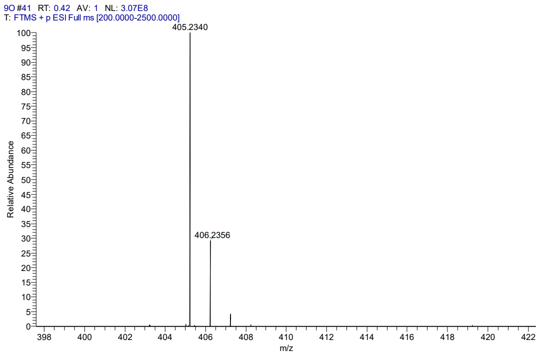

^{31}PNMR of **3-8**(C_9OH)

HRMS of **3-8** (C_9OH)

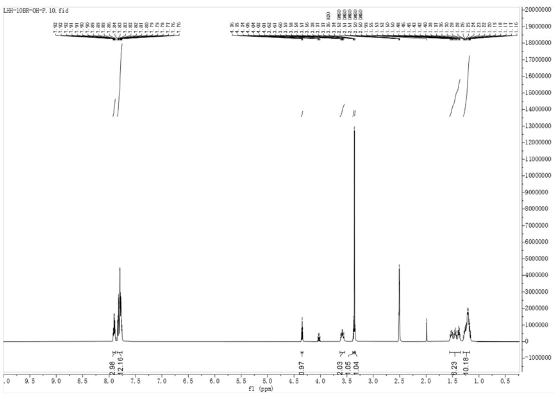

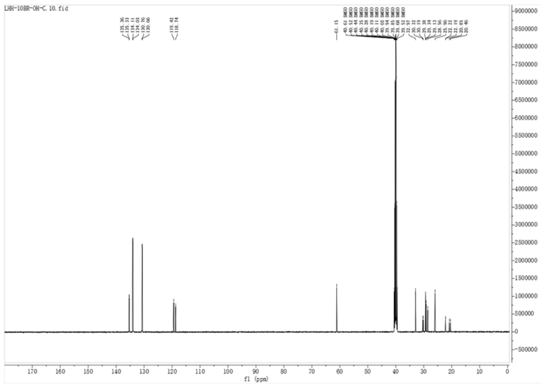

^{1}HNMR of **3-9** ($C_{10}OH$)

^{13}CNMR of **3-9**($C_{10}OH$)

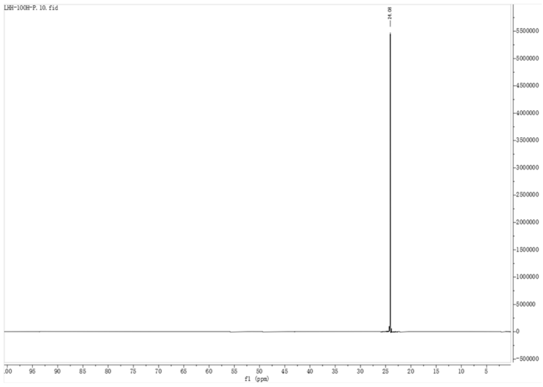

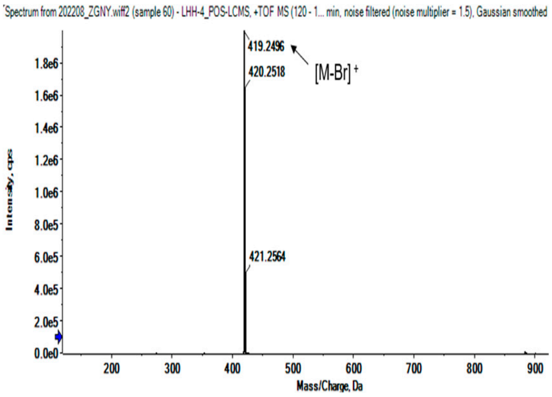

^{31}PNMR of **3-9**($C_{10}OH$)

HRMS of **3-9** ($C_{10}OH$)

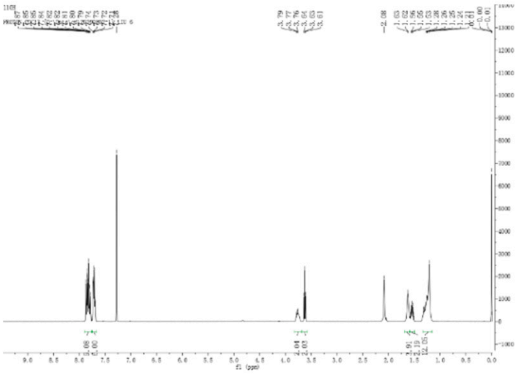

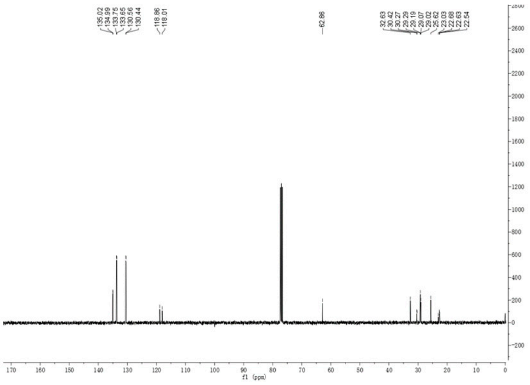

^{1}HNMR of **3-10** ($C_{11}OH$)

^{13}CNMR of **3-10** ($C_{11}OH$)

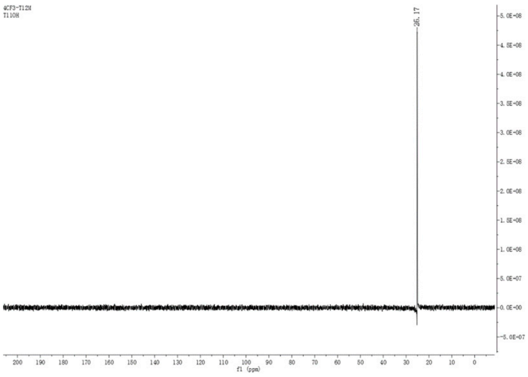

^{31}PNMR of **3-10** ($C_{11}OH$)

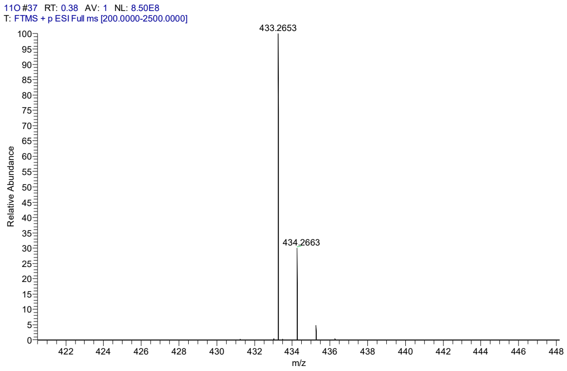

HRMS of **3-10** ($C_{11}OH$)

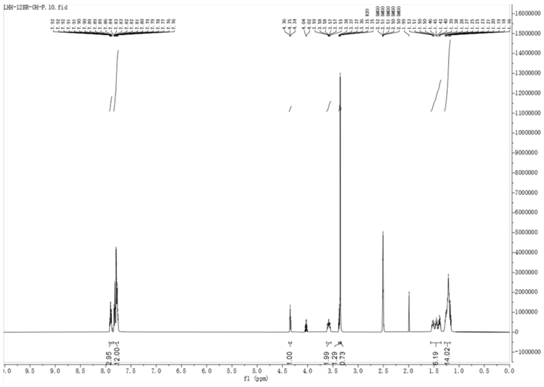

^{1}HNMR of **3-11** ($C_{12}OH$)

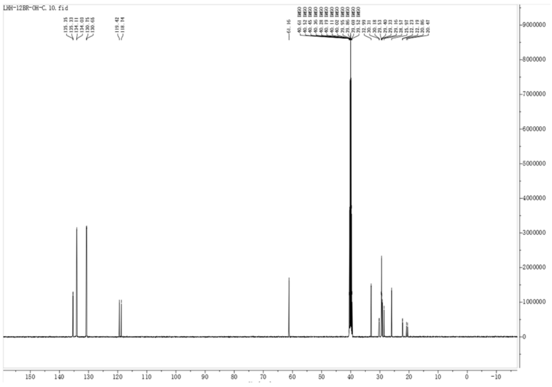

^{13}CNMR of **3-11**($C_{12}OH$)

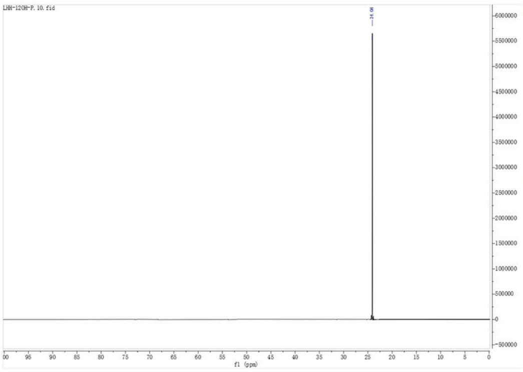

^{31}PNMR of **3-11**($C_{12}OH$)

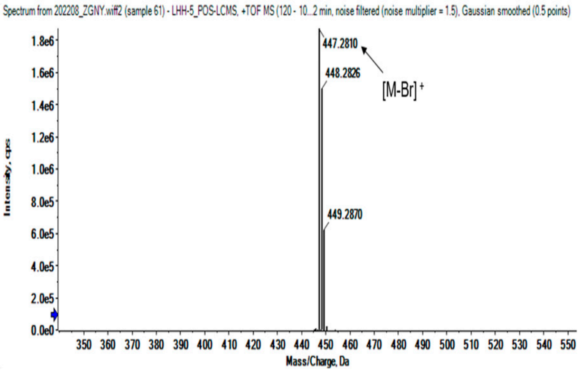

HRMS of **3-11** ($C_{12}OH$)

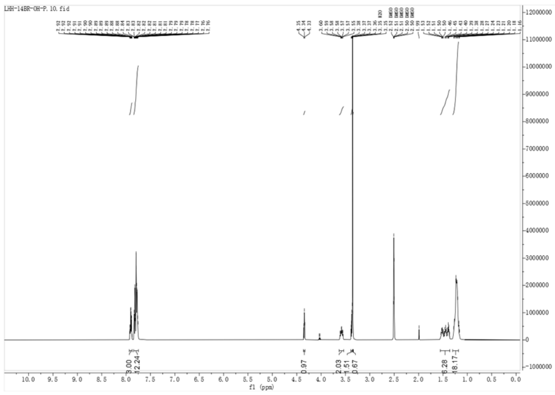

^{1}HNMR of **3-12** ($C_{14}OH$)

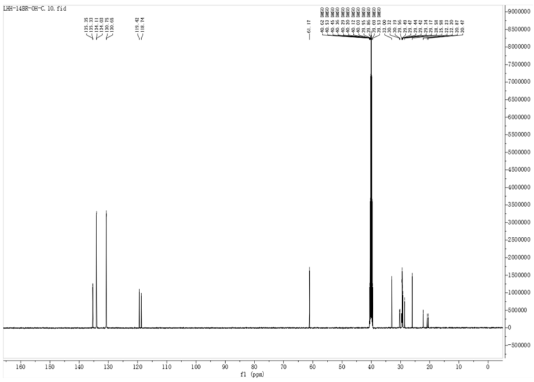

^{13}CNMR of **3-12**($C_{14}OH$)

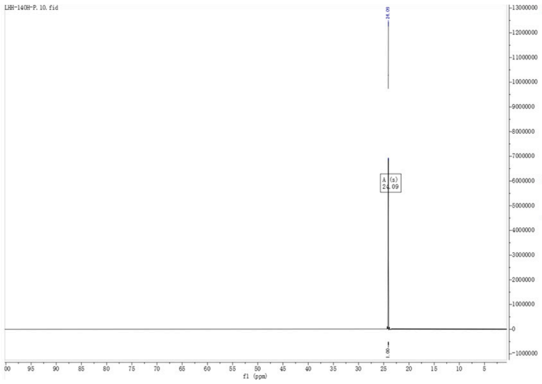

^{31}PNMR of **3-12**($C_{14}OH$)

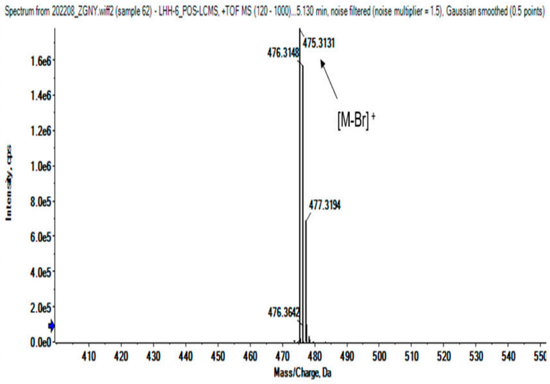

HRMS of **3-12** ($C_{14}OH$)

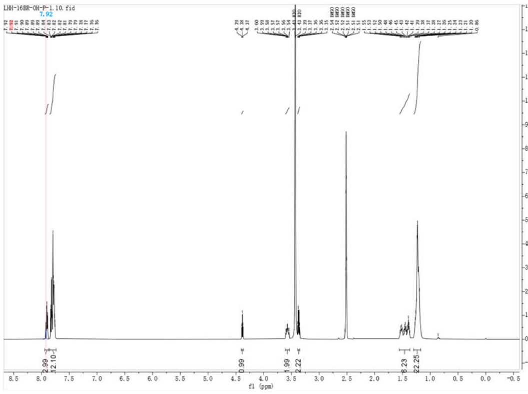

^{1}HNMR of **3-13** ($C_{16}OH$)

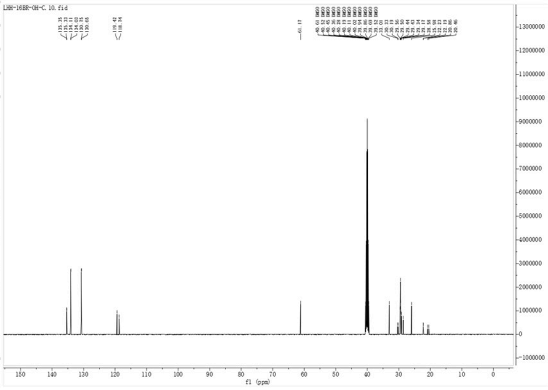

^{13}CNMR of **3-13**($C_{16}OH$)

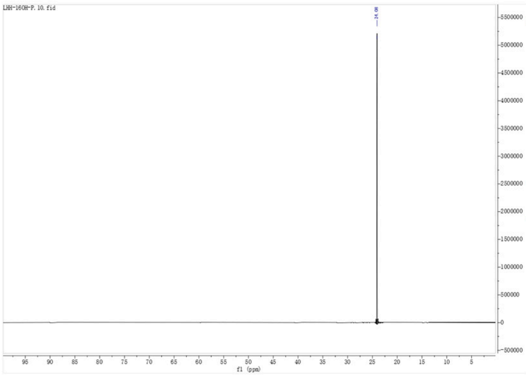

^{31}PNMR of ($C_{16}OH$)

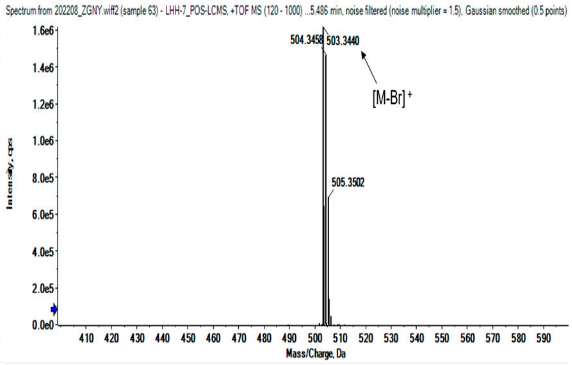

HRMS of **3-13** ($C_{16}OH$)

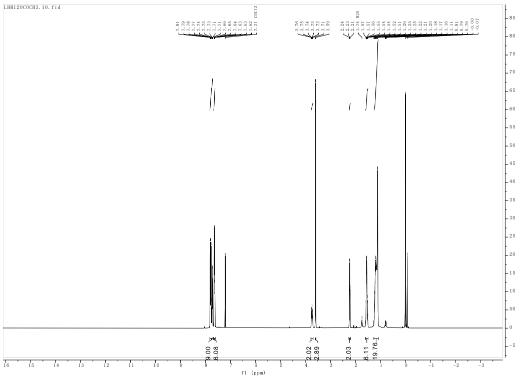

^{1}HNMR of 2-12′

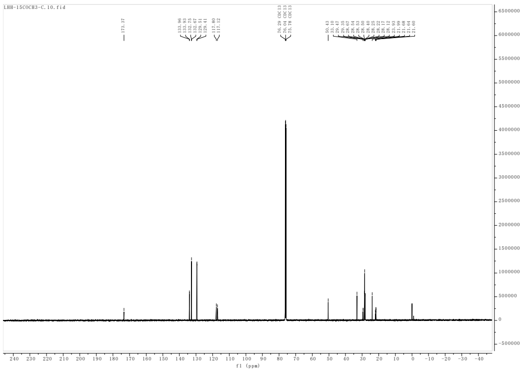

^{13}CNMR of 2-12′

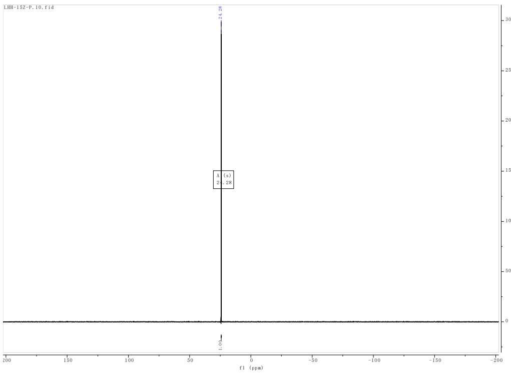

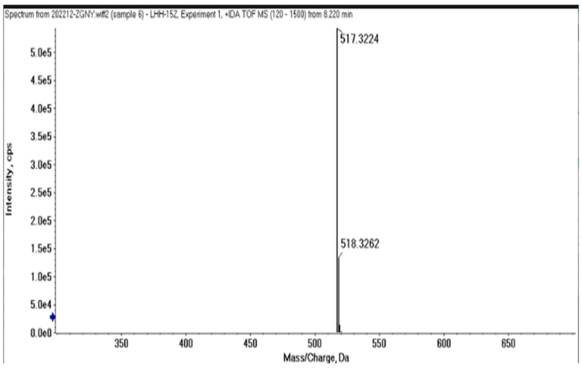

^{31}PNMR of 2-12′

HRMS of 2-12′

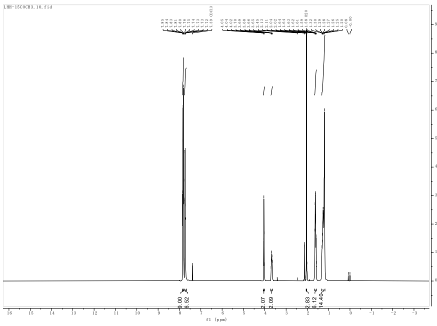

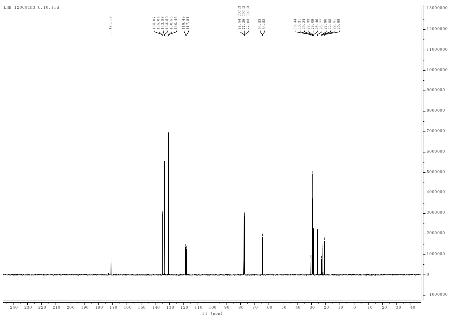

^{1}HNMR of 3-11′

^{13}CNMR of 3-11′

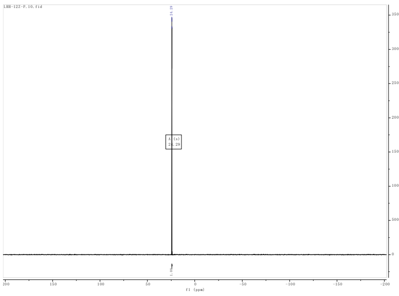

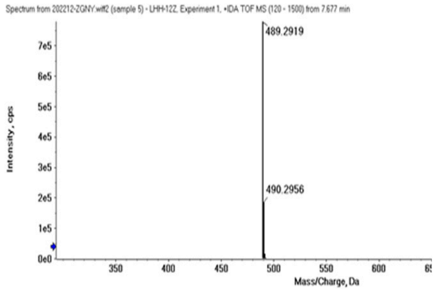

^{31}PNMR of 3-11′

HRMS of 3-11′

Table S1.

Table S1. Relative permeability of *P. capsici* mycelia after treatment with agents (%)

Compd.	1h	3h	5h	7h	9h
DMSO	4.71±3.71	14.63±2.43	20.49±2.05	23.17±0.81	24.79±2.49
fluazinam	18.21±5.45	37.59±3.54	40.92±4.26	45.07±2.42	45.55±3.66
**1-8**	15.65±1.18	27.37±2.34	31.85±2.94	35.53±3.16	37.67±1.73

**2-12**	7.50±0.84	15.01±1.96	17.54±0.90	21.21±1.58	20.94±1.81
**3-11**	12.15±1.66	23.15±0.26	26.18±0.69	26.41±0.87	27.83±0.16

References

(1) Liu, X. L.; Yang, D. Y.; Yin, F. H.; Li, J.-Q.; Xiao, Y. M.; Fu, B.; Qin, Z. H. The application of "Plug-in molecules" method in novel strobilurin fungicides screening. *RSC Adv.* **2020**, *10*, 42804−42809.

(2) Kang, Z.; Gu, B. G. Standard Operating Practice for Pesticide Biological Activity Test-Fungicide Roll; Chemical Industry Press: Beijing, **2016**; pp 1−206.

(3) Hughes, J. K.; Hodge, A.; Fitter, A. H.; Atkin, O. K. Mycorrhizal respiration: implications for global scaling relationships. *Trends Plant Sci.* **2008**, *13*, 583−588.

(4) Cai, G.; Yu, W.; Song, D.; Zhang, W.; Guo, J.; Zhu, J.; Ren, Y.; Kong, L. Discovery of fluorescent coumarin-benzo[b]thiophene 1,1-dioxide conjugates as mitochondria-targeting antitumor STAT3 inhibitors. *Eur. J. Med. Chem.* **2019**, *174*, 236−251.

(5) Yin, X. D.; Ma, K. Y.; Wang, Y. L.; Sun, Y.; Shang, X. F.; Zhao, Z. M.; Wang, R. X.; Chen, Y. J.; Zhu, J. K.; Liu, Y. Q. Design, synthesis, and antifungal evaluation of 8-hydroxyquinoline metal complexes against phytopathogenic fungi. *J. Agric. Food Chem.* **2020**, *68*, 11096−11104.
